# Simultaneous expression of three G genotypes of VP7 proteins in a recombinant porcine rotavirus confers protective immunity against multiple rotavirus infections

**DOI:** 10.1128/jvi.02015-25

**Published:** 2026-03-18

**Authors:** Xi Cheng, Hui Deng, Xianyu Bian, Jianxin Wang, Chen Wang, Nan Han, Jinzhu Zhou, Xuejiao Zhu, Xuehan Zhang, Xiaojing Yang, Ran Tao, Bin Li

**Affiliations:** 1 College of Veterinary Medicine, Nanjing Agricultural University 261674, Nanjing, Jiangsu, China; 2 Institute of Veterinary Medicine, Key Laboratory of Veterinary Biological Engineering and Technology, Ministry of Agriculture and Rural Affairs, Jiangsu Key Laboratory for Food Quality and Safety-State Key Laboratory Cultivation Base of Ministry of Science and Technology, Jiangsu Academy of Agricultural Sciences 668638 , Nanjing, Jiangsu, China; 3 Jiangsu Co-Innovation Center for the Prevention and Control of Important Animal Infectious Diseases and Zoonoses, Jiangsu Key Laboratory of Zoonoses, Yangzhou University 38043, Yangzhou, Jiangsu, China; 4 GuoTai (Taizhou) Center of Technology Innovation for Veterinary Biologicals, Taizhou, Jiangsu, China; University of Michigan Medical School, Ann Arbor, Michigan, USA

**Keywords:** porcine rotavirus, reverse genetics system, multivalent recombinant virus, VP7, vaccine

## Abstract

Porcine rotavirus (PoRV) is a significant pathogen that causes diarrhea in piglets, with the G9, G5, and G4 genotypes being the most prevalent in China. Although vaccination is the most effective strategy to prevent PoRV infections, the currently available G5 genotype-based vaccine offers limited cross-protection against other circulating PoRV genotypes in pig farms. In this study, we developed an entirely plasmid-based reverse genetics (RG) system for the PoRV strain NJ2012 (G9P[7]) and generated two recombinant reporter viruses expressing the fluorescent UnaG and NLuc proteins, respectively. Furthermore, we successfully constructed multivalent recombinant PoRV strains by inserting the VP7 gene of G4 genotype into the gene segment 7 (NSP3) and/or the VP7 gene of G5 genotype into the gene segment 5 (NSP1) within the backbone of rNJ2012-WT strain. These multivalent recombinant viruses efficiently expressed G4 and/or G5 genotype of the VP7 protein in infected cells and elicited robust immune responses in mice. The adult mice immunized with the trivalent recombinant PoRV (rNJ2012-fG5-VP7/haG4-VP7), which simultaneously expressed VP7 proteins from G4, G5, and G9 genotypes, conferred passive protection to suckling mice against infections caused by multiple G genotypes of PoRV. In summary, these findings established a platform for efficient generation of multivalent recombinant PoRV, offering a scalable methodology to facilitate the development of next-generation PoRV vaccines.

**IMPORTANCE:**

Porcine rotavirus (PoRV) is a primary etiological agent of diarrhea in swine, posing significant challenges due to the diversity of circulating genotypes and the limited cross-protection offered by existing PoRV vaccines. To address this, we developed multivalent recombinant vaccine candidates capable of eliciting robust immunity against multiple genotypes of PoRV strains. Using the established entirely plasmid-based reverse genetics (RG) system based on the G9 genotype of PoRV strain NJ2012 (G9P[7]), we further engineered the multivalent recombinant virus simultaneously expressing VP7 proteins from G4, G5, and G9 genotypes. Immunization of adult mice with this trivalent recombinant virus conferred broad-spectrum passive protection to their suckling mice against infections by multiple PoRV genotypes. Our findings established a novel platform for efficiently developing multivalent PoRV vaccines, offering a promising strategy for the prevention and control of PoRV outbreaks.

## INTRODUCTION

Rotavirus (RV) is a leading cause of viral diarrhea in newborns and young animals worldwide, which severely damage human health and animal husbandry industry (1, 2). As a member of the *Sedoreoviridae* family and *Rotavirus* genus, RV possesses a genome comprising 11 double-stranded RNA segments, encoding 6 structural proteins (VP1–VP4, VP6, VP7) and 6 non-structural proteins (NSP1–NSP6) (3). RVs are classified into groups A-J based on the antigenicity and genetic characteristics of the outer capsid protein VP6 (4). Group A Rotavirus A (RVA) is the predominant strain responsible for its high prevalence and pathogenicity (5, 6). Besides, the G and P genotyping system was established based on the two outer capsid proteins, VP7 and VP4, which are known to elicit neutralizing antibodies (7, 8). These properties underscore the importance of VP7 and VP4 as fundamental antigens in the development of RV vaccines, with the VP7 protein being especially prominent (9, 10). For instance, due to the limited cross-protection among different genotypes, current human RV vaccines primarily employ multivalent G-type formulations (11).

Porcine rotavirus (PoRV), first isolated from diarrheic piglets in 1976 (12), has emerged as a globally prevalent pathogen, causing substantial economic losses in the swine industry (2). In China, PoRV was the second most detected virus in diarrheic pig samples, with reported detection rates ranging from 16.8% to 72.77% (13–16). Additionally, PoRV has been shown to enhance the pathogenicity of porcine epidemic diarrhea virus (PEDV) (17). Epidemiological studies have identified 12 G genotypes and 18 P genotypes in swine, with G9 being the dominant genotype (>80%), followed by G5 and G4 (13, 15, 16, 18). Despite this diversity, the only commercially available PoRV vaccine (included in a trivalent vaccine against TGEV, PEDV, and PoRV) remains based on the G5 genotype (19). Given the limited cross-protection between genotypes, there is an urgent need for broad-spectrum multivalent PoRV vaccines capable of addressing the circulating strain diversity.

The plasmid-based reverse genetics (RG) system, as one of the critical tools for viral engineering and vaccine development, has been extensively utilized since its establishment in 2017 (20). This system has enabled the generation of diverse recombinant RVs across multiple genotypes and species, including human, murine, and bovine (10, 20–24). It has also facilitated the development of reporter RVs that express fluorescent proteins (UnaG, eGFP, luciferase) (25, 26) and the creation of novel vector-based vaccines that incorporate antigens from various pathogens (the severe acute respiratory syndrome coronavirus 2 [SARS-CoV-2] spike epitopes, human norovirus [HuNoV] VP1, and HuNoV P protein) (27–30). Despite the widespread prevalence of G9 PoRV, no RG systems or applications for this genotype have been reported.

In this study, we established an efficient RG system for G9 genotype of PoRV based on the PoRV NJ2012 strain (G9P[7]). Two fluorescent reporter viruses that encode UnaG or NLuc from NSP3 gene construct (rNJ2012-NSP3-UnaG and rNJ2012-NSP3-NLuc) were generated by using the system. Furthermore, three multivalent recombinant viruses expressing G4 genotype of VP7 (insertion of the NSP3 gene) and/or G5 genotype of VP7 (insertion of the NSP1 gene) proteins were engineered (rNJ2012-NSP3-haG4-VP7, rNJ2012-NSP1-fG5-VP7, and rNJ2012-fG5-VP7/haG4- VP7). Mice immunized with multivalent recombinant viruses can effectively elicit immune responses and confer cross-protection against infections caused by multiple PoRV strains in suckling mice.

## RESULTS

### Establishment of the RG system for G9P[7] genotype PoRV strain NJ2012

**Fig 1:**
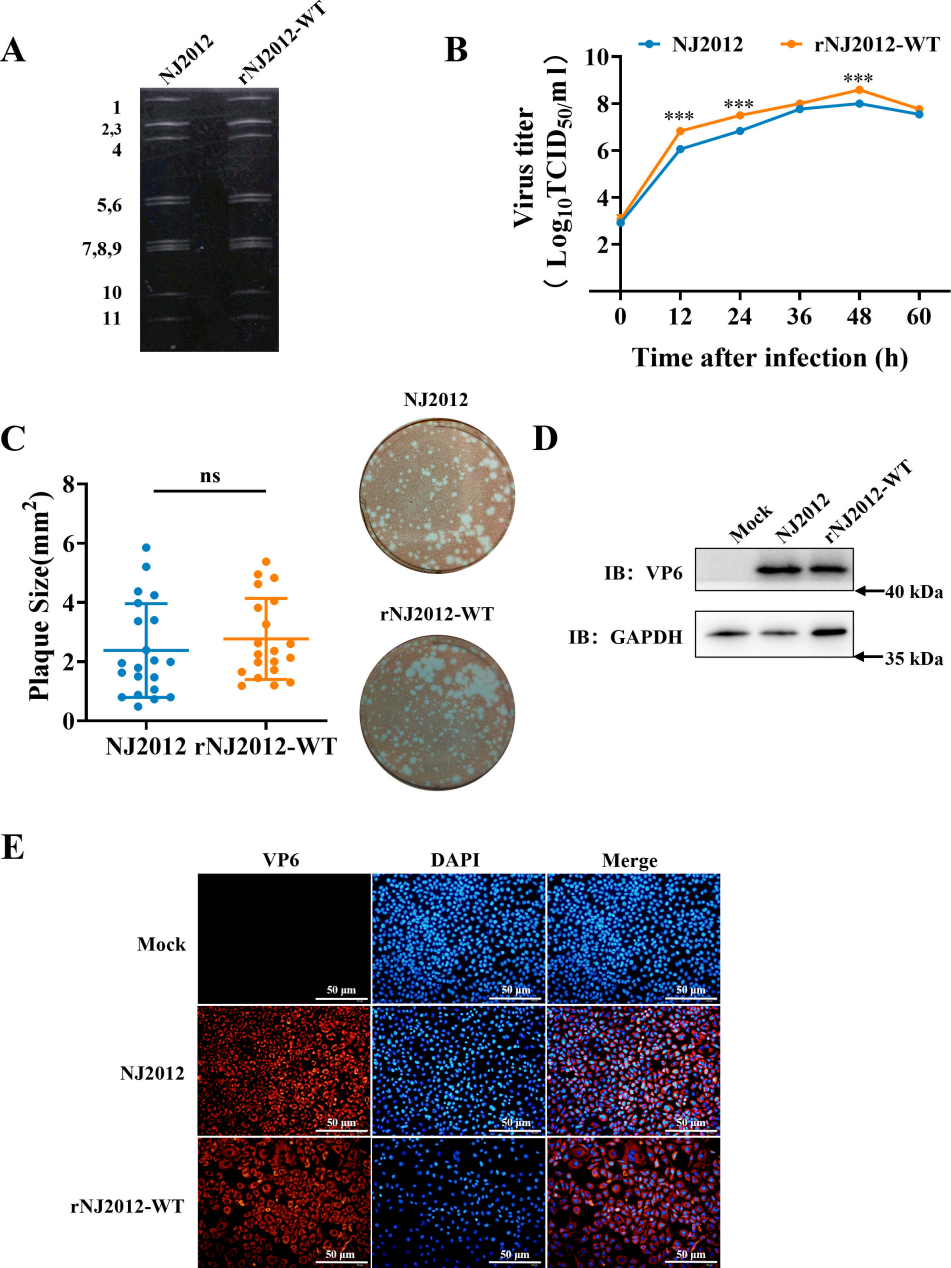
Establishment of the RG system for G9P[7] genotype PoRV strain NJ2012. (**A**) dsRNA profiles. Viral dsRNA genomes were isolated from purified virions, and genome segments 1–11 of viruses are indicated on the left side of the panels. (**B**) Replication kinetics. Viral samples were snap-frozen at each designated time point, and viral titers were determined through TCID_50_ analysis and plotted as curves. Statistical analysis was performed using Student’s *t*-test for each time point. Statistical significance is represented as follows: ***P < 0.001. (**C**) Comparison of plaque size. Representative photographs of viral plaques are shown. The sizes of at least 20 randomly selected plaques from two independent plaque assays were measured using ImageJ and reported as area in relative units. Statistical analysis was performed using Student’s *t*-test. Statistical significance is represented as follows: ns, not significant. (**D**) Western blotting analysis. Protein expression of RV-VP6 and GAPDH was detected using specific antibodies. (**E**) Immunofluorescence assays. The cells were stained with antibodies specific to RV-VP6 (red) and DAPI (blue).

To generate the recombinant PoRV strain rNJ2012-WT (G9P[7]), we transfected 11 sequence-verified pT7-NJ2012 plasmids into BHK-T7 cells. Recombinant viruses were then passaged and amplified in MA104 cells, with their dsRNA profiles analyzed via gel electrophoresis. Electrophoretic analysis confirmed that the dsRNA migration patterns between rNJ2012-WT and parental NJ2012 were identical (Fig. 1A). Both replication kinetics and plaque assays demonstrated that rNJ2012-WT exhibited biological characteristics comparable to those of its parental virus (Fig. 1B and C), confirming its efficient replication in MA104 cells (Fig. 1D and E). These findings validate the successful rescue of the G9 genotype recombinant PoRV strain rNJ2012-WT.

### Generation of the recombinant NJ2012 strain expressing UnaG or NLuc fluorescent protein

**Fig 2:**
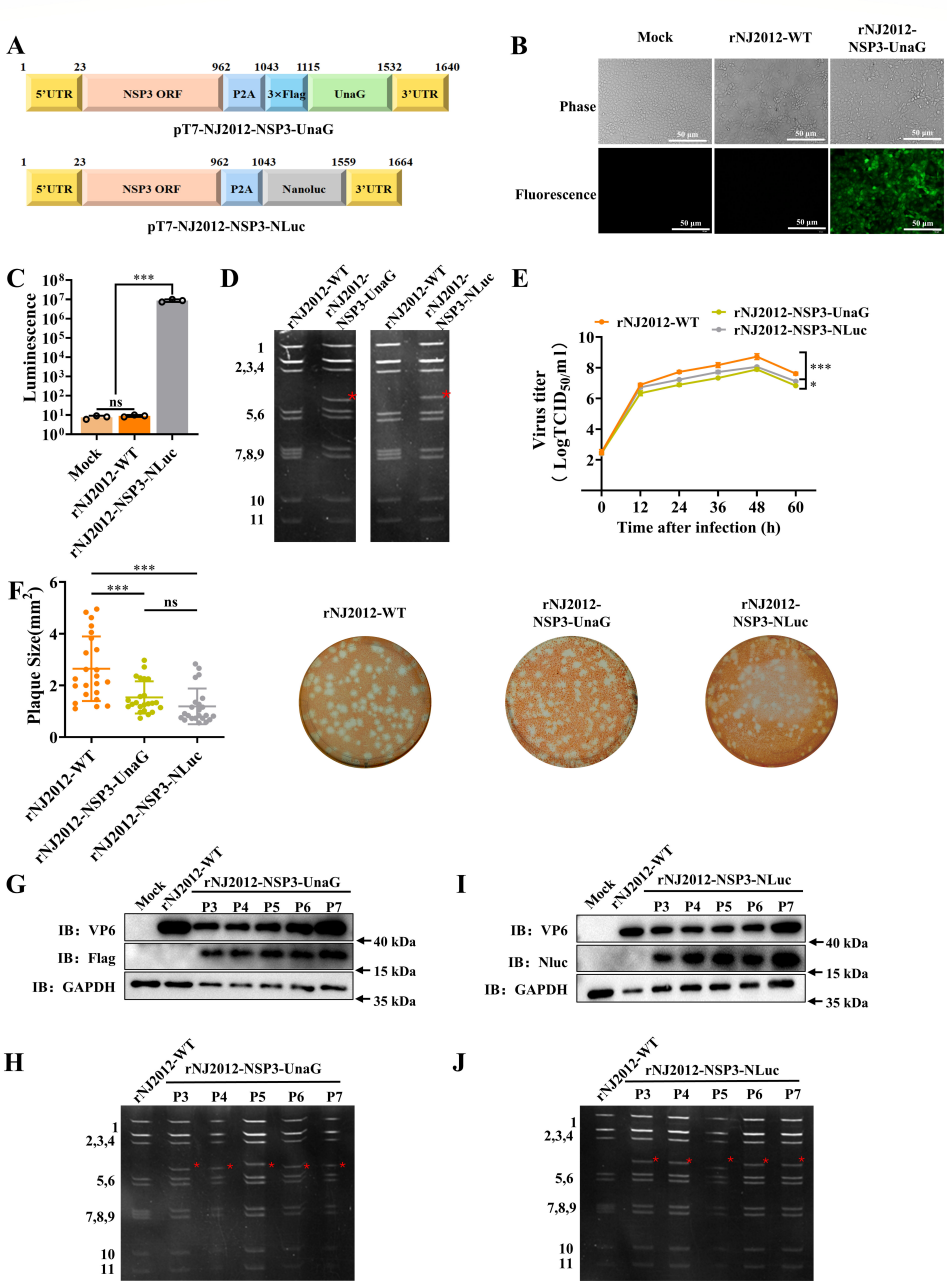
Generation of the recombinant NJ2012 strain expressing UnaG or NLuc fluorescent protein. (**A**) A schematic diagram (not to scale) of the pT7 plasmid engineered to express UnaG and NLuc, indicating nucleotide positions. (**B**) Fluorescence detected in MA104 cells that were infected by rNJ2012-WT or rNJ2012-NSP3-UnaG using fluorescence microscopy. (**C**) Luciferase activity of rNJ2012-WT and rNJ2012-NSP3-NLuc. Statistical analysis was performed using Student’s *t*-test for results expressed as the mean luminescence of triplicates. Statistical significance is represented as follows: ***P < 0.001. (**D**) dsRNA profiles. The migrations of modified genome segment 7 are indicated by a red asterisk. Genome segments 1–11 of recombinant viruses are indicated on the left side of the panels. (**E**) Replication kinetics. Viral samples were snap-frozen at each designated time point, and viral titers were determined through TCID_50_ analysis and plotted as curves. Statistical analysis was performed using Student’s *t*-test for each time point. Statistical significance is represented as follows: *P < 0.05 and ***P < 0.001. (**F**) Comparison of plaque size. Representative photographs of viral plaques are shown. The sizes of at least 20 randomly selected plaques from two independent plaque assays were measured using Image J and reported as area on relative units. Statistical analysis was performed using Student’s *t*-test. Statistical significance is represented as follows: ns, not significant, and ***P < 0.001. (**G and H**) Genetic stability of rNJ2012-NSP3-UnaG. Western blotting analysis demonstrated that the UnaG gene retained functionality within the viral genome, and the recombinant virus preserved *in vitro* infectivity and genetic stability. The migrations of the modified genome segment 7 of viral dsRNAs are indicated with a red arrow. Genome segments 1–11 of the recombinant viruses are indicated on the left side of the panels. (**I and J**) Genetic stability of rNJ2012-NSP3-NLuc. Western blotting analysis demonstrated that the NLuc gene remained functional within the viral genome, and the recombinant virus retained *in vitro* infectivity and genetic stability. The migrations of modified genome segment 7 of viral dsRNAs are indicated with a red arrow. Genome segments 1–11 of recombinant viruses are indicated on the left side of the panels.

Reporter viruses engineered to express fluorescent proteins are invaluable tools for fluorescence-based imaging assays in RV research. In this study, we investigated the feasibility of generating recombinant NJ2012 virus that expresses fluorescent protein within the segment 7 RNA. We engineered pT7-NJ2012-NSP3-UnaG and pT7-NJ2012-NSP3-NLuc plasmids harboring either the UnaG or NLuc gene within the PoRV gene segment 7 (Fig. 2A). Using the RG system, we successfully rescued recombinant PoRV strains rNJ2012-NSP3-UnaG and rNJ2012-NSP3-NLuc. Live-cell imaging revealed robust UnaG fluorescence in rNJ2012-NSP3-UnaG-infected cells compared to rNJ2012-WT (Fig. 2B). Luciferase assays confirmed the functional expression of NLuc in rNJ2012-NSP3-NLuc-infected cells (Fig. 2C). The expression of the reporter genes in infected MA104 cells was validated through western blotting and immunofluorescence assays (IFA) (Fig. S1). NLuc was detected using an anti-NLuc antibody, while UnaG was identified with anti-Flag antibodies due to methanol fixation-induced fluorescence quenching. Modified segment 7 dsRNA exhibited reduced electrophoretic mobility due to insertions (Fig. 2D). Although both reporter viruses exhibited slightly reduced titers compared to rNJ2012-WT at 24–60 h post-infection (hpi), they continued to replicate efficiently in MA104 cells (Fig. 2E). Plaques formed by rNJ2012-WT were larger than those formed by rNJ2012-NSP3-UnaG and rNJ2012-NSP3-NLuc (Fig. 2F). Genetic stability was confirmed over five serial passages in MA104 cells. Western blotting and dsRNA-PAGE analyses of two recombinant viruses through serial passages (P3–P7) revealed no significant differences in viral proteins or genome profiles (Fig. 2G through J). These results collectively demonstrate that both rNJ2012-NSP3-UnaG and rNJ2012-NSP3-NLuc maintained stable fluorescent protein expression across at least five serial passages.

### Generation of multivalent recombinant NJ2012 strain simultaneously expressing G4 and/or G5 genotypes of VP7 proteins

**Fig 3:**
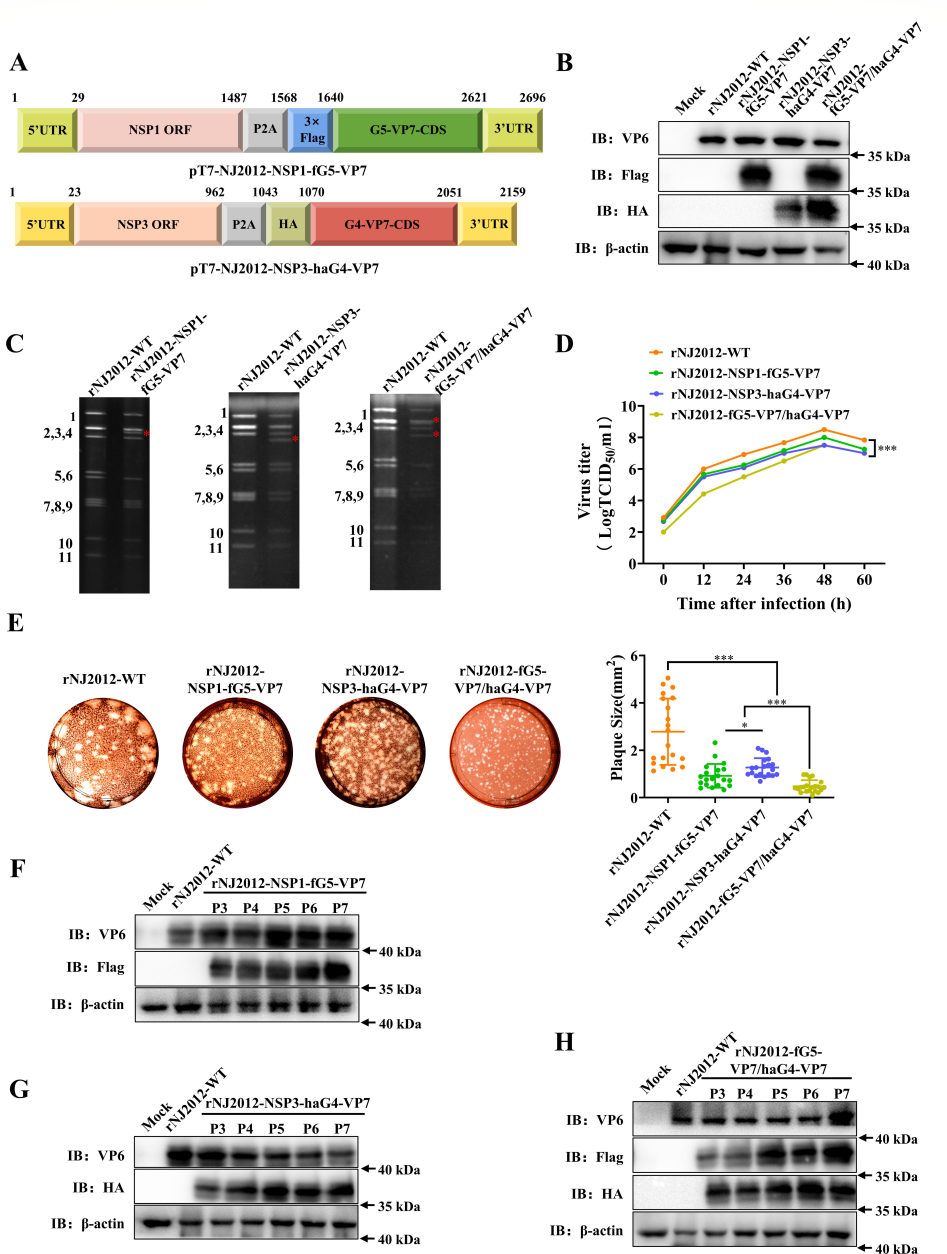
Generation of multivalent recombinant NJ2012 strain simultaneously expressing G4 and/or G5 genotypes of VP7 proteins. (**A**) A schematic diagram (not to scale) of the pT7 plasmid engineered to express G4-VP7 and G5-VP7, indicating nucleotide positions. (**B**) Western blotting analysis. Protein expression of RV-VP6, Flag, HA, and GAPDH was detected by the specific antibodies. (**C**) dsRNA profiles. The migrations of modified genome segment 5 and genome segment 7 are indicated by a red asterisk. Genome segments 1–11 of recombinant viruses are indicated on the left side of the panels. (**D**) Replication kinetics. Viral samples were snap-frozen at each designated time point, and viral titers were determined through TCID_50_ analysis and plotted as curves. Statistical analysis was performed using Student’s *t*-test for each time point. Statistical significance is represented as follows: ***P < 0.001. (**E**) Comparison of plaque size. Representative photographs of viral plaques are shown. The sizes of at least 20 randomly selected plaques from two independent plaque assays were measured using ImageJ and reported as area on relative units. Statistical analysis was performed using Student’s *t*-test. Statistical significance is represented as follows: *P < 0.05 and ***P < 0.001. (**F–H**) Genetic stability of rNJ2012-NSP1-fG5-VP7, rNJ2012-NSP3-haG4-VP7, and rNJ2012-fG5-VP7/haG4-VP7. Western blotting analysis confirmed that both G4-VP7 and G5-VP7 retained functionality within the viral genome, and the recombinant virus retained *in vitro* infectivity and genetic stability.

The potential of utilizing NJ2012 as a platform for developing multivalent vaccines was subsequently explored. To generate rNJ2012-fG5-VP7/haG4-VP7, we constructed pT7-NSP1-fG5-VP7 that expresses VP7 protein of JSNJ2024 (G5) in PoRV gene segment 5 and pT7-NSP3-haG4-VP7 that expresses VP7 protein of JSJR2023 (G4) in PoRV gene segment 7 (Fig. 3A). To overcome the cross-reactivity of VP7 antibodies, HA and Flag epitope tags were incorporated at the N-termini of G4-VP7 and G5-VP7 for unambiguous detection, respectively. Subsequently, rNJ2012-NSP1-fG5-VP7, rNJ2012-NSP3-haG4-VP7, and rNJ2012-fG5-VP7/haG4-VP7 were successfully rescued using the RG system. Western blotting and IFA confirmed epitope expression in rNJ2012-fG5-VP7/ haG4-VP7-infected MA104 cells (Fig. 3B; Fig. S2). The modified segments 5 and 7 exhibited reduced electrophoretic mobility due to insertions (Fig. 3C). Replication kinetics resembled the parental strain, but quantitative analysis confirmed attenuated *in vitro* replication following foreign protein insertion (Fig. 3D) (22, 26). Despite modest reductions in titer at 24–60 hpi compared to rNJ2012-WT, all recombinant strains exhibited robust replication. Plaque assays revealed that rNJ2012-WT produced larger plaques than rNJ2012-NSP1-fG5-VP7, rNJ2012-NSP3-haG4-VP7, and rNJ2012-fG5-VP7/haG4 -VP7 (Fig. 3E), consistent with the kinetics observed in reporter viruses. Western blotting and dsRNA-PAGE confirmed the genetic stability of all three recombinant viruses through serial passages (P3–P7) in MA104 cells, with stable expression of both G4 and G5 genotypes of VP7 proteins across all recombinant strains (Fig. 3F through H; Fig. S3). Collectively, these results established NJ2012 as a promising candidate vector for developing multivalent vaccines.

### The multivalent recombinant PoRV expressing multiple genotypes of VP7 elicited a more efficient immune response than the wild-type strain in mice

**Fig 4:**
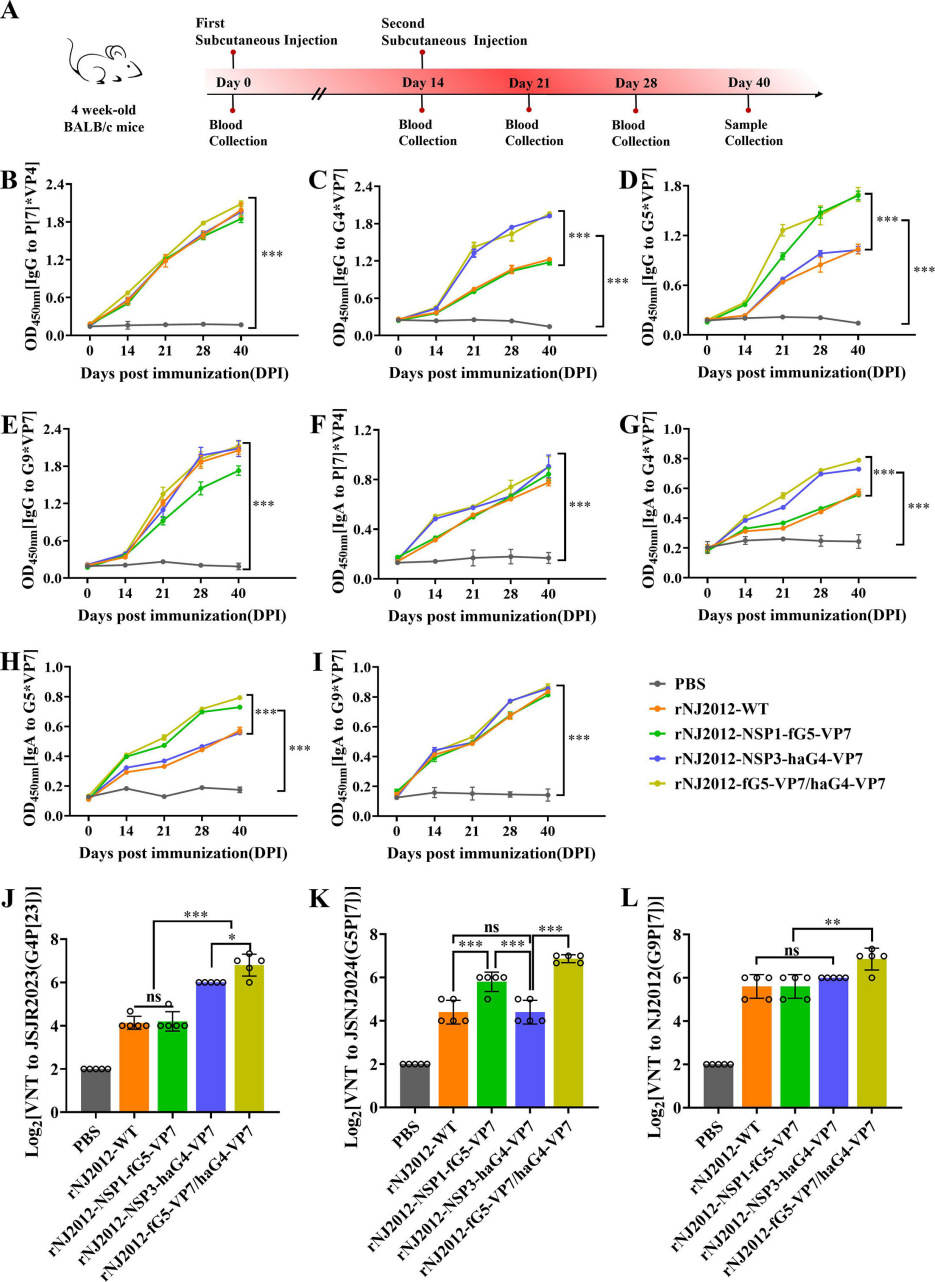
The multivalent recombinant PoRV expressing multiple genotypes of VP7 elicited a more efficient immune response than the wild-type strain in mice. (**A**) Schematic diagram of the active immunization model in BALB/c mice. (**B–E**) Mouse sera were serially diluted and tested in ELISA for anti-P[7]-VP4 IgG, anti-G4-VP7, anti-G5-VP7, or anti-G9-VP7. Statistical analysis was performed using two-way ANOVA. Statistical significance is represented as follows: ***P < 0.001. (**F–I**) The amount of fecal IgA against P[7]-VP4, G4-VP7, G5-VP7, and G9-VP7 in fecal suspension from BALB/c mice was determined by ELISA. Statistical analysis was performed by two-way ANOVA. Statistical significance is represented as follows: ***P < 0.001. (**J–L**) Serum neutralization activities against PoRV genotypes G4, G5, and G9. Mouse sera were serially diluted, mixed with PoRV, and incubated at 37°C for 1 h. Statistical analysis was performed using Student’s *t*-test. Statistical significance is represented as follows: ns, not significant; *P < 0.05; **P < 0.01; ***P < 0.001.

To evaluate the immunogenicity of rNJ2012-NSP1-fG5-VP7, rNJ2012-NSP3-haG4-VP7, and rNJ2012-fG5-VP7/haG4-VP7, four-week-old BALB/c mice were subcutaneously immunized with 10^6^ TCID_50_ of either recombinant viruses or wild-type rNJ2012-WT. Blood and stool specimens were collected at 14, 21, 28, and 40 days post-immunization (dpi) (Fig. 4A). Enzyme-linked immunosorbent assay (ELISA) was utilized to quantify specific immunoglobulin G (IgG) levels for P[7]-VP4, G4-VP7, G5-VP7, and G9-VP7 across various time points. Serum samples from all immunized groups showed significantly higher IgG titers against all target antigens compared to the PBS control group at every time point assessed. Remarkably, the G4/5/9 trivalent-vaccinated group demonstrated the most robust antibody response (Fig. 4B through E). Secretory immunoglobulin A (sIgA) in the intestinal lumen serves as a critical defense mechanism against enteric pathogens (27, 31). To assess whether rNJ2012-fG5-VP7/haG4-VP7 elicits mucosal immunity following subcutaneous immunization, we quantified PoRV-specific intestinal immunoglobulin A (IgA) in fecal samples. Consistent with previous studies (27, 32, 33), immunized mice exhibited detectable RV-specific fecal IgA (Fig. 4F through I). We further evaluated serum neutralizing activity against G4, G5, and G9 genotypes of PoRV. The G4/5/9 trivalent-vaccinated group induced neutralizing antibodies against all three genotypes, while the G4/9 and G5/9 bivalent groups generated neutralizing antibodies targeting their respective genotypes; the G9 monovalent group elicited antibodies specific only to the G9 genotype (Fig. 4J through L). In summary, these data demonstrate that rNJ2012-fG5-VP7/haG4-VP7 induced significant mucosal and systemic immune responses against G4, G5, and G9 genotypes of PoRV in mice.

**Fig 5:**
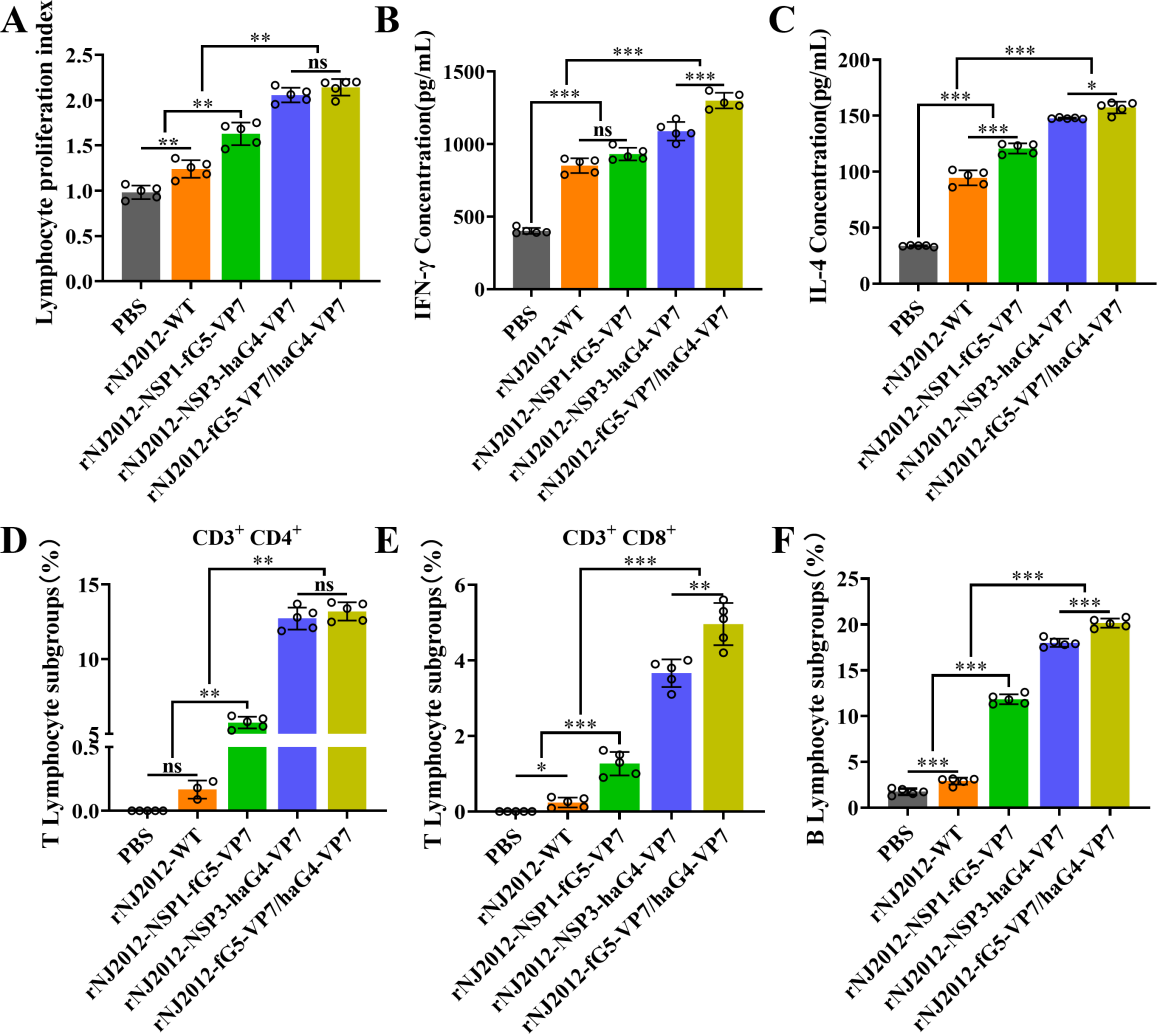
Cellular immune responses in BALB/c mice immunized with recombinant G9 PoRV strains expressing VP7 proteins of G4 and/or G5 genotypes. (**A**) Isolation of splenocytes for lymphocyte proliferation assay. (**B and C**) Quantification of IFN-γ and IL-4 secreted by splenocyte supernatants from immunized mice through ELISA. (**D–F**) Flow cytometry analysis of the proportions of CD3+CD4+ T lymphocytes, CD3+CD8+ T lymphocytes, and B cells in splenocytes from different experimental groups. Statistical analysis was performed using Student’s *t*-test. Statistical significance is represented as follows: ns, not significant; *P < 0.05; **P < 0.01; ***P < 0.001.

Splenocytes were isolated from mice at 40 dpi to assess T-cell reactivity against the trivalent recombinant virus. The rNJ2012-fG5-VP7/haG4-VP7 construct elicited significantly stronger lymphocyte proliferation compared to the other immunized groups (Fig. 5A). ELISA quantification of interferon gamma (IFN-γ) and interleukin-4 (IL-4) in splenocyte supernatants revealed that the trivalent vaccine induced significantly higher production of IFN-γ and IL-4 production than all other immunized groups (Fig. 5B and C). Flow cytometry analysis consistently reflected these patterns in T-cell and B-cell populations alongside cytokine production profiles (Fig. 5D through F; Fig. S4). Collectively, these data demonstrated that the trivalent recombinant virus effectively enhances cellular immunity through robust antigen-specific T-cell proliferation, pronounced Th1/Th2 cytokine polarization, and synchronized lymphocyte activation.

### Passive immunization of rNJ2012-fG5-VP7/haG4-VP7 significantly protected suckling mice against G4, G5, and G9 genotypes of PoRV infections

**Fig 6:**
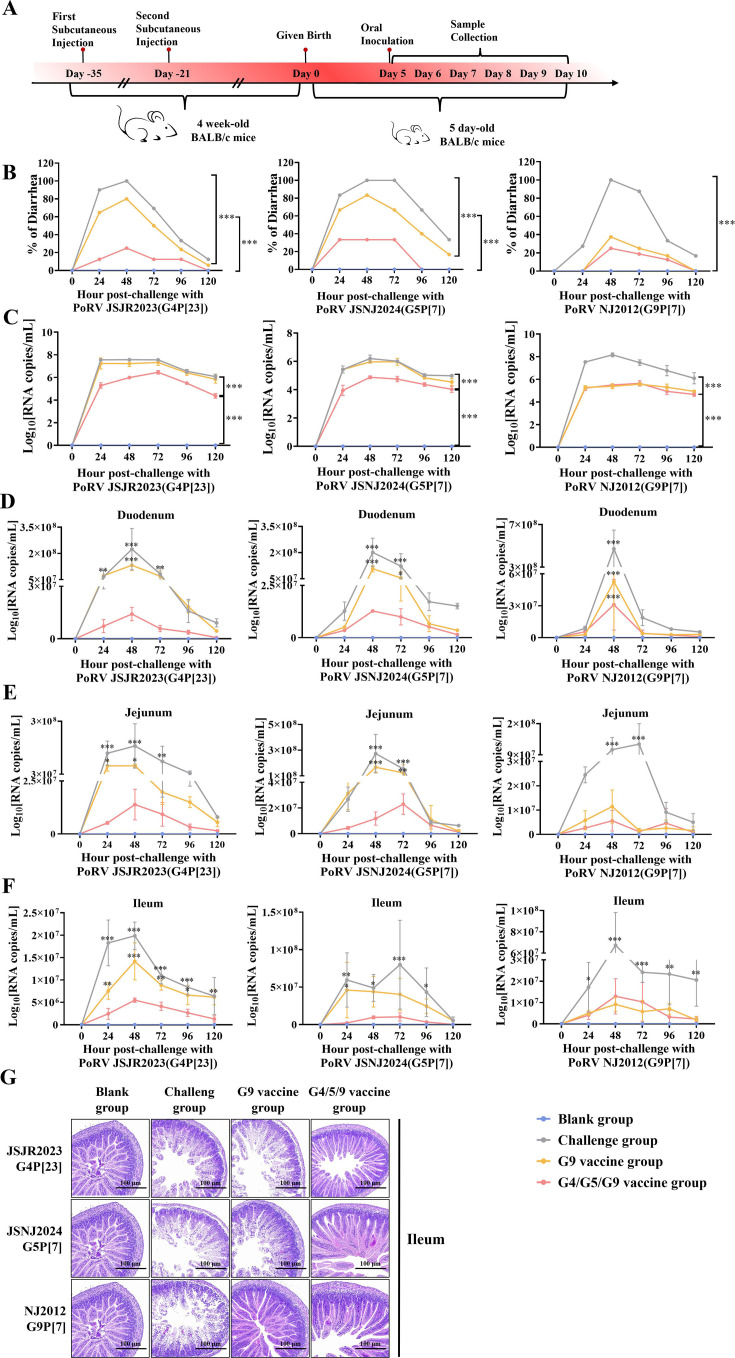
Passive immunization of rNJ2012-fG5-VP7/haG4-VP7 significantly protected suckling mice against G4, G5, and G9 genotypes of PoRV infections. (**A**) Schematic diagram of the passive immunization model in BALB/c suckling mice. (**B**) Cohort-specific diarrhea dynamics in BALB/c mice following PoRV JSJR2023, JSNJ2024, or NJ2012 challenge. (**C**) Group-specific quantification of RV shed in stool samples by RT-qPCR. (**D–F**) Group-specific quantification of RV duodenal, jejunal, and ileal viral load by RT-qPCR. Statistical analysis was performed using two-way ANOVA. Statistical significance is represented as follows: ns, not significant; *P < 0.05; **P < 0.01; ***P < 0.001. (**G**) Histopathological lesions of ileum lesions from suckling mice challenged with PoRV JSJR2023 (G4P[23]), JSNJ2024 (G5P[7]), or NJ2012 (G9P[7]).

The results from the active immunization of three multivalent recombinant viruses in mice demonstrated that rNJ2012-fG5-VP7/haG4-VP7 exhibited significantly greater efficacy compared to the other two viruses. To further evaluate the protective efficacy of the trivalent recombinant virus in suckling mice, we conducted a passive immunization study. Female BALB/c mice were subcutaneously immunized twice with 10^6^ TCID_50_ of rNJ2012-fG5-VP7/haG4-VP7 or wild-type rNJ2012-WT, after which they were cohoused with male mice. Following a three-week gestation period, five-day-old suckling mice were divided into several groups and orally challenged with JSJR2023 (G4P[23]), JSNJ2024 (G5P[7]), or NJ2012 (G9P[7]) (Fig. 6A). The blank group consisted of suckling mice born to PBS-treated female mice and was not subjected to viral challenge. The viral challenge group comprised suckling mice born to PBS-treated female mice that were subsequently challenged with the viruses. The G9 vaccine group included suckling mice born to female mice immunized with rNJ2012-WT and exposed to viral challenge, whereas the G4/5/9 trivalent-vaccinated group consisted of suckling mice born to dams immunized with rNJ2012-fG5-VP7/haG4-VP7 and subsequently challenged with the viruses. All challenged groups developed diarrhea from 1 to 5 days post-challenge, with clinical signs peaking at 48 h post-challenge during which significant weight loss (*P* < 0.001) and maximal viral shedding were observed. In contrast, suckling mice from the G4/5/9 trivalent-vaccinated group exhibited significantly reduced incidence of diarrhea and lower levels of viral shedding compared to the challenged control group (Fig. 6B and C). Subsets of challenged neonates were euthanized at 24-hour intervals to collect intestinal samples (duodenum, jejunum, and ileum) until terminal necropsy was performed at 120 h. Real-time quantitative PCR (RT-qPCR) was used to quantify daily viral loads (Fig. 6D through F). The challenged group showed significantly higher intestinal viral loads than all other groups (*P* < 0.001). Furthermore, suckling mice from the G4/5/9 trivalent-vaccinated group exhibited substantially lower viral loads than those in the G9 vaccine group. Histopathological analysis confirmed characteristic epithelial denudation of the small intestinal villi in the challenge group (Fig. 6G), while no significant intestinal lesions were observed in either the blank group or the G4/5/9 trivalent-vaccinated group. Additionally, pathological changes were absent in the G9 vaccine group when infected NJ2012. These results demonstrated that immunization with rNJ2012-fG5-VP7/haG4-VP7 simultaneously provided passive protection in suckling mice against infections caused by G4, G5, and G9 genotypes of PoRV.

### The rNJ2012-fG5-VP7/haG4-VP7 transferred immune response from adult mice to suckling mice

**Fig 7:**
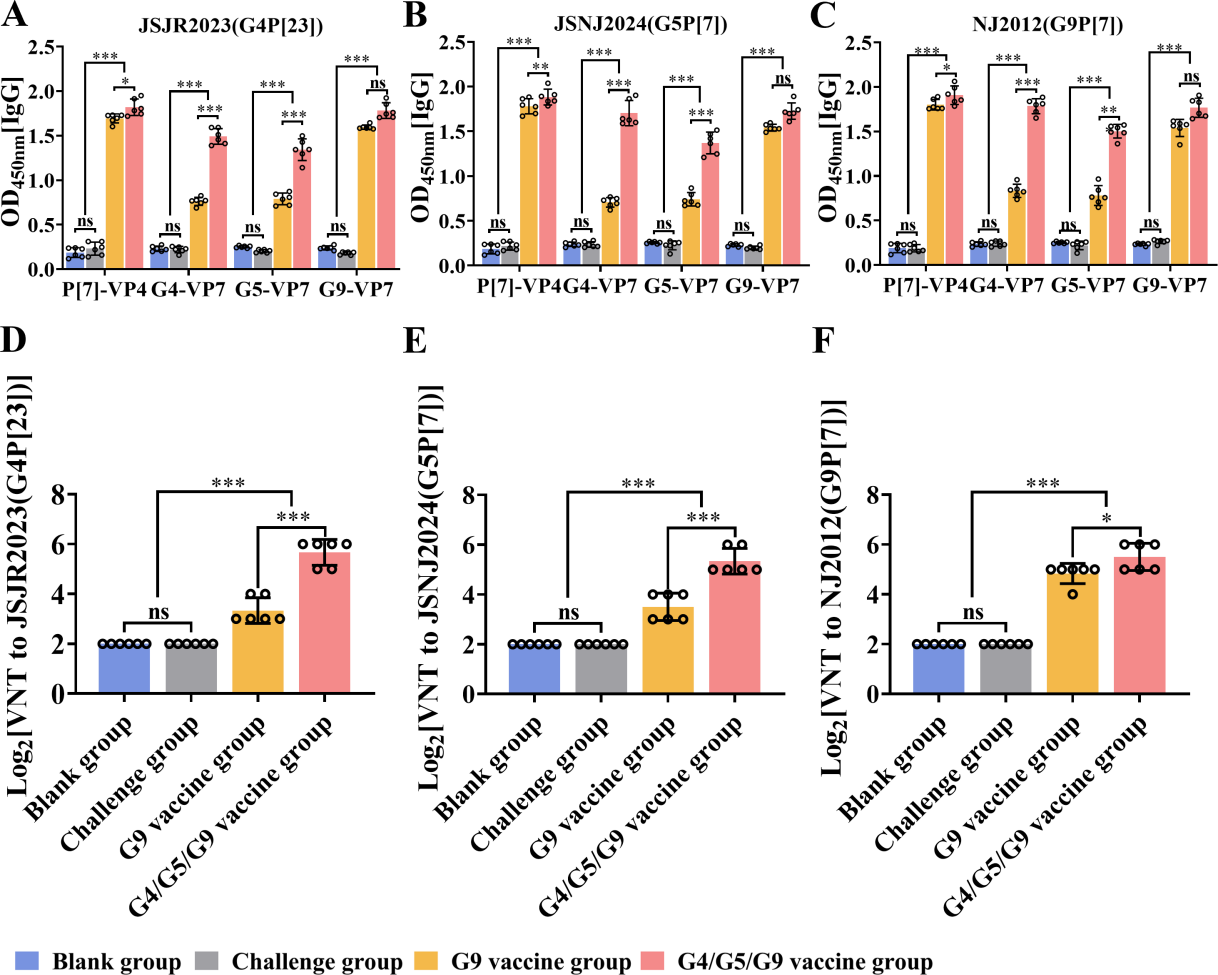
The rNJ2012-fG5-VP7/haG4-VP7 transferred immune response from adult mice to suckling mice. (**A–C**) Mouse sera were serially diluted and tested in ELISA for anti-P[7]-VP4 IgG, anti-G4-VP7, anti-G5-VP7, or anti-G9-VP7. Statistical analysis was performed using two-way ANOVA. Statistical significance is represented as follows: ns, not significant; *P < 0.05; **P < 0.01; ***P < 0.001. (**D–F**) Serum neutralization activities against G4, G5, and G9 genotypes of PoRV. Statistical analysis was performed using Student’s *t*-test. Statistical significance is represented as follows: ns, not significant; *P < 0.05; ***P < 0.001.

The passive immunity derived from vaccinating pregnant animals plays a critical role in protecting newborns from the significant economic burdens associated with RV diarrhea outbreaks (34–36). Therefore, a key factor in determining the practical application of a vaccine is its ability to confer passive protection to offspring through maternal immunization. To evaluate maternally derived serum IgG antibody levels in suckling mice, we measured ELISA binding titers against P[7]-VP4, G4-VP7, G5-VP7, and G9-VP7 across various experimental groups. Suckling mice born to females vaccinated with the G4/5/9 trivalent vaccine displayed elevated IgG levels, whereas negligible IgG was detected in both blank and challenged groups (Fig. 7A through C). To further evaluate the presence of maternally derived neutralizing antibodies, we assessed serum neutralization activity against G4, G5, and G9 genotypes of PoRV. Suckling mice from G4/5/9 trivalent-vaccinated adult mice exhibited the highest neutralizing antibody titers against G4, G5, and G9 genotypes of PoRV, showing levels exceeding 2^5^ (Fig. 7D and F). These findings demonstrated the effective passive transfer of antibodies from immunized adult mice to their suckling offspring through lactation. Overall, the experimental results demonstrated that rNJ2012-fG5-VP7/haG4-VP7 elicited robust genotype-specific antibodies in suckling mice, which were efficiently transmitted via maternal immunity.

## DISCUSSION

**Fig 8:**
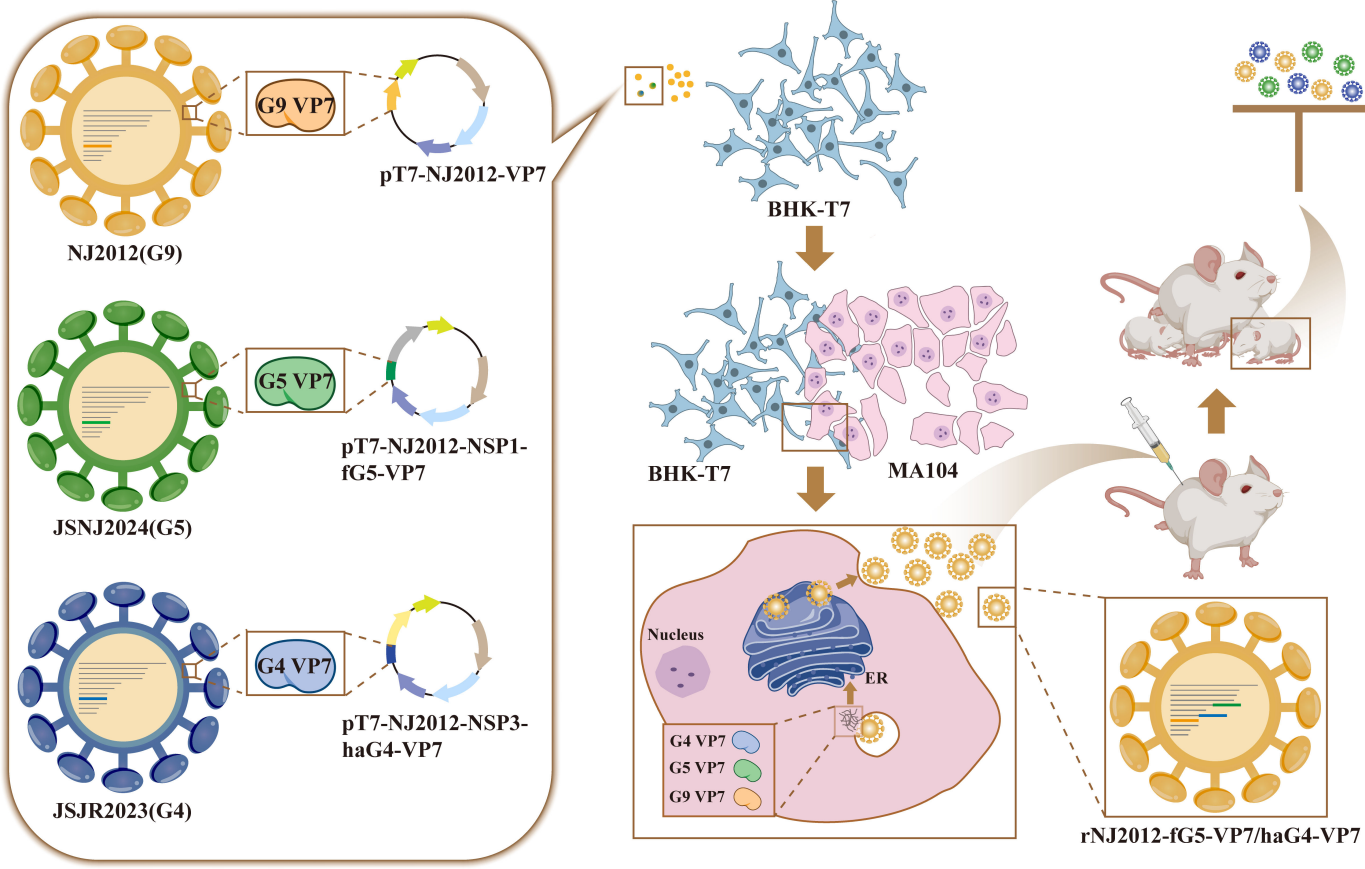
Schematic model of the construction of the trivalent recombinant viruses and their passive immune-protective efficacy in suckling mice. The multivalent recombinant virus simultaneously expressing VP7 proteins from G4, G5, and G9 genotypes was engineered utilizing the RG system. Immunization of adult mice with this multivalent recombinant virus induced broad-spectrum passive protection to suckling mice against infections caused by multiple genotypes of PoRV.

PoRV has attracted significant attention in recent years due to its high detection rates and increasing genotype complexity (6). Current G5 genotype vaccines provided limited cross-protection against diverse genotypes of PoRV in pigs. Therefore, the development of multivalent vaccines that confer broader protection against circulating PoRV strains is urgently needed. In this study, we established the RG system for G9P[7] genotype PoRV strain NJ2012 and exploited permissive insertion sites within the NSP1 and NSP3 open reading frames (ORFs) to permit stable heterologous gene expression (25, 37). Using this platform, we engineered fluorescent reporter viruses and developed multivalent vaccine candidates. Murine immunization and passive-protection models demonstrated that the G4/5/9 trivalent vaccine elicited robust immunogenicity and conferred broad-spectrum passive protection against three G genotypes of PoRV infections in suckling mice (Fig. 8). These findings offer novel strategic insights and a conceptual foundation for next-generation PoRV vaccines and therapeutics.

Using the established RG system, we engineered two fluorescent reporter viruses, rNJ2012-NSP3-UnaG and rNJ2012-NSP3-NLuc, both of which retained genetic and functional stability through five serial passages (Fig. 2G). Aside from a slightly smaller plaque size compared to the parental strain (Fig. 2F), the two reported viruses displayed *in vitro* biological properties comparable to the parental strain, including viral protein expression and replication (Fig. 2E; Fig. S1). This observation differs from previously reported outcomes for similar recombinant viruses (20, 24, 26), suggesting that NJ2012 may have an enhanced capacity to accommodate exogenous proteins. Collectively, these results highlighted NJ2012 as a promising vector for exogenous protein expression and underscored its substantial potential for future research and vaccine development.

Given the central role of VP7 in eliciting immune responses, we engineered recombinant G9 PoRV strains expressing VP7 proteins from the G4 and G5 genotypes to develop multivalent PoRV vaccines. Notably, the peak titers of rNJ2012-fG5-VP7/haG4-VP7 were over 100-fold lower than those of rNJ2012-WT (Fig. 3D). These results suggest that the concurrent insertion of exogenous genes into both NSP1 and NSP3 might impose limitations on RV replication. Indeed, recombinant RVs engineered as plug-and-play expression platforms through modifications to the NSP3 and/or NSP1 ORF often exhibit lower growth rates compared to their parental strain (20, 26–28, 30). The VP7 protein is essential for the stable assembly of virus particles due to its interactions with other structural proteins, such as VP4 (38). It is possible that variations in interaction mechanisms among VP7 proteins from different G genotypes could affect their expression and assembly efficiency. Based on the study by Philip and Kawagishia (27, 30), we anticipated that rNJ2012-fG5-VP7/haG4-VP7 would stably express VP7 proteins from the G4 and G5 genotypes. Although all three G genotype VP7 proteins may theoretically be displayed on the virus surface simultaneously, the complexity of virus assembly and the interactions between heterologous genes may impede effective expression. To better understand the underlying mechanisms, further experiments, such as immunoelectron microscopy analysis, are warranted. *In vivo* experiments showed that the trivalent recombinant virus rNJ2012-fG5-VP7/haG4-VP7 induced a substantially stronger humoral and cellular immune response compared to the two bivalent recombinant viruses, rNJ2012-NSP1-fG5-VP7 and rNJ2012-NSP3-haG4-VP7 (Fig. 4B through E, 4J through L, 5A and B). We hypothesized that the immunogenicity of the trivalent recombinant virus may be independent of its replication efficiency, attributable to the concurrent expression of multiple G genotype VP7 proteins. This expression likely stimulated the activation of intestinal B cells (39, 40), leading to a diverse antibody response capable of neutralizing the pathogen.

Previous studies have indicated that the size of exogenous genes is directly correlated with their genomic stability during RV replication. The potential strategies to enhance the genetic stability of these exogenous genes include incorporating extended sequences from the 3′ untranslated region (UTR) of RV (41) or replacing full-length fragments with key neutralizing antigenic epitopes (27). These strategies present opportunities for future optimization. For instance, substituting the full-length VP7 segment with key neutralizing antigenic epitopes from domain I of VP7 could reduce the size of the expressed foreign protein (42). This reduction may minimize its impact on viral proliferation, thus facilitating the development of a next-generation broad-spectrum vaccine that is more stable, exhibits higher expression efficiency, and ensures better cross-reactivity. Alternatively, incorporating additional strains representing different G genotypes into this platform could provide cross-protection against multiple RV genotypes.

As a multivalent recombinant virus capable of simultaneously expressing VP7 proteins from the G4, G5, and G9 genotypes, rNJ2012-fG5-VP7/haG4-VP7 presents several advantages. This approach employs a safe and efficient vector to carry a recombinant virus expressing multiple G genotypes of proteins, thereby simplifying the integration of a multivalent vaccine. Currently, most human RV vaccines are designed using RV as the backbone to generate multivalent viruses. For instance, RotaTeq contains five genotypes of RVs (G1, G2, G3, G4, and P[8]) derived from both human and animal sources (11). The multivalent recombinant virus not only reduces production costs but also enhances the safety of vaccine manufacturing by circumventing risks such as viral contamination inherent to traditional production methods. Additionally, it elicits robust humoral and cellular immune responses that provide protection to suckling mice against G4, G5, and G9 genotypes of PoRV infections. These findings highlight the potential of this recombinant virus as a promising candidate for PoRV vaccination.

However, further studies are needed to obtain deeper insights into the immunogenicity and protective efficacy of rNJ2012-fG5-VP7/haG4-VP7. Although the data obtained from murine models are encouraging, future evaluation of the protective immunity of these recombinant strains in pigs is essential. While murine models are widely used to investigate PoRV immunogenicity and passive antibody-mediated protection (43, 44), they do not replicate the development of long-term immunological memory, which is crucial for ensuring durable vaccine efficacy. Moreover, the current experimental design primarily focuses on assessing systemic antibody levels, including serum antibody titers and neutralizing antibodies, offering limited insights into intestinal immune responses, particularly IgA secretion and immune cell activity in suckling mice. Given that rotavirus infections predominantly occur in the intestines (36), these mucosal immune mechanisms are critical for providing effective protection. Additionally, the platform employed in this study is capable of co-expressing two heterologous proteins, allowing for the construction of recombinant viruses that can express VP4 proteins from prevalent PoRV P genotypes, such as P[13] and P[23]. Moreover, the platform can be adapted to express immunostimulatory peptides, broadening protection against a wider range of RV genotypes. Furthermore, clinical trials have demonstrated that the live-attenuated oral RV vaccine exhibited a high efficacy (97%) against severe gastroenteritis caused by RVs (11). Based on these findings, we propose the development of a single-round infectious variant of NJ2012 that lacks genes essential for viral particle assembly, offering a safe and effective candidate for oral vaccination (45–47). This strategy could facilitate the generation of a candidate vaccine library by modifying existing safe and effective licensed PoRV vaccines. In conclusion, the data presented in this study hold significant practical implications, providing valuable insights for the design of RV-based multivalent vaccine candidates that may target not only RVs but also other prevalent viral, bacterial, and parasitic pathogens.

## MATERIALS AND METHODS

### Cell lines and viruses

MA104 cells (*Cercopithecus aethiops* embryo kidney epithelial cells) and BHK-21 cells (Baby hamster kidney cells) stably expressing T7 RNA polymerase (BHK-T7) were cultured in Dulbecco’s Modified Eagle Medium (DMEM, BasalMedia, China), supplemented with 10% Fetal Bovine Serum (FBS, Bionic, Israel). The PoRV strains NJ2012 (G9P[7], GenBank: MT874983.1–MT874993.1), JSJR2023 (G4P[23], GenBank: PP100149.1–PP100159.1), and JSNJ2024 (G5P[7]) were isolated and maintained in our laboratory (48, 49). All the PoRV strains were propagated in MA104 cells cultured in DMEM supplemented with 0.5 µg/mL trypsin (Sigma-Aldrich, USA).

### Plasmid construction

The pT7 plasmid and three helper plasmids pCAG-D1R, pCAG-D12L, and pCAG-FAST-p10 were generously contributed by Dr. Takeshi Kobayashi of Osaka University (originally obtained from Addgene)(20). The helper plasmid C3P3-G1 was generously contributed by Dr. Siyuan Ding at the University of Washington (50). Both pCAGGS-HA-NSP2 and pCAGGS-HA-NSP5 were constructed and maintained in our laboratory (49).

Full-length cDNAs encompassing all 11 segments of the NJ2012 dsRNA genome were reverse-transcribed and amplified from parental viral RNA extracts. These amplicons were subsequently cloned into T7 promoter-driven rescue plasmids to enable genomic RNA transcription under T7 RNA polymerase control. This process generated the full complement of rescue vectors pT7-NJ2012-VP1, pT7-NJ2012-VP2, pT7-NJ2012-VP3, pT7-NJ2012-VP4, pT7-NJ2012-VP6, pT7-NJ2012-VP7, pT7- NJ2012-NSP1, pT7-NJ2012-NSP2, pT7-NJ2012-NSP3, pT7-NJ2012-NSP4, and pT7-NJ2012-NSP5. Following previously described approaches (25, 26), pT7-NJ2012-NSP3-UnaG and pT7-NJ2012-NSP3-NLuc plasmids were produced by fusing DNA fragments for P2A-3×Flag UnaG or P2A-NLuc, respectively, to the 3′ end of the NJ2012-NSP3 ORF via homologous recombination. To generate the plasmid harboring VP7 protein for JSJR2023 (G4) in RV gene 7 (pT7-NJ2012-NSP3-haG4-VP7), the HA-tagged gene encoding the G4-VP7 sequence was amplified by 2× KeyPo SE Master Mix (Vazyme, China) and replaced the sequence encoding UnaG in the pT7-NJ2012-NSP3-UnaG. Based on the capability of the RV NSP1 segment to accommodate heterologous genes (51), we inserted a P2A peptide-encoding sequence together with the Flag-tagged VP7 gene derived from the PoRV strain JSNJ2024 (G5) into the 3′ end of the NJ2012-NSP1 ORF to generate the plasmid pT7-NJ2012-NSP1-fG5-VP7. The primer sequences used for plasmid construction are listed in Table S1. All plasmid sequences were verified by Sanger DNA sequencing and purified using the OMEGA plasmid purification kit (OMEGA, USA).

### Generation of recombinant PoRV

For the rescue of recombinant viruses, a total of 0.4 µg each of pT7-NJ2012-VP1, pT7-NJ2012-VP2, pT7-NJ2012-VP3, pT7-NJ2012-VP4, pT7-NJ2012-VP6, pT7-NJ2012-VP7, pT7-NJ2012-NSP1, pT7-NJ2012-NSP3, pT7-NJ2012-NSP4, C3P3-G1, pCAG-D1R, pCAG-D12L, pCAGGS-HA-NSP2, and pCAGGS-HA-NSP5; 0.8 µg each of pT7-NJ2012-NSP2 and pT7-NJ2012-NSP5; and 15 ng of pCAG-FAST-p10 were combined in 125 µL Opti-MEM (Thermo Fisher Scientific, USA) with 25 µL TransIT-LT1 transfection reagent (Mirus Bio, USA) and transfected into BHK-T7 cells. To facilitate the expression of exogenous proteins in the NJ2012 backbone, pT7-NJ2012-NSP1 and/or pT7-NJ2012-NSP3 plasmids were replaced with modified plasmids encoding the corresponding modified NSP1 and/or NSP3. After 2 days post-transfection, 5 × 10⁵ MA104 cells were added to each well. After 3 days of co-culture, the clarified supernatant was collected and then inoculated onto MA104 cells. CPE was monitored at 24, 48, and 72 hpi.

### NLuc luciferase assay

MA104 cells were infected in triplicate with either parental or recombinant viruses at multiplicity of infection (MOI) of 0.05 for 12 h and then added to 50 µL/well of Nano-Glo Luciferase Assay Reagent (Promega, USA). All procedures were carried out according to the manufacturer’s instructions.

### Immunoblot assays

MA104 cells infected with parental and recombinant viruses at an MOI of 0.05 were lysed at 12 hpi using PMSF-supplemented RIPA buffer (Beyotime, China). The primary antibodies and their corresponding dilutions were as follows: Flag (Proteintech, 1:10,000) rabbit polyclonal antibody and HA (Proteintech, 1:10,000), VP6 (made in lab, 1:10,000) (19), and anti-β-actin (Proteintech, 1:10,000) mouse monoclonal antibody. The bound primary antibodies were detected using 1:10,000 dilutions of horseradish peroxidase (HRP)-conjugated secondary antibodies (goat anti-mouse IgG [Solarbio, China] and goat anti-rabbit IgG [Solarbio, China]) in PBST. HRP signals were developed using the SuperSignal West Pico PLUS Chemiluminescent substrate (Thermo Fisher Scientific, USA) and visualized using a Tanon imaging system (Tanon, China).

### Immunofluorescence assay

MA104 cells were infected with either parental or recombinant viruses at an MOI of 0.05. At 12 hpi, cells were fixed with absolute ethanol. Immunofluorescence staining was performed using primary antibodies: anti-Flag (1:1,000) rabbit polyclonal antibody, anti-VP6 (1:1,000), and anti-HA (1:1,000) mouse monoclonal antibody. These were followed by secondary antibodies: Cy3-conjugated goat anti-mouse IgG (Bosterbio, 1:250) and FITC-conjugated goat anti-rabbit IgG (Bosterbio, 1:250). Nuclei were counterstained with DAPI (Beyotime, 1:10,000). Images were acquired using fluorescence microscopy.

### Electrophoresis of viral dsRNA genomes

Double-stranded RNA (dsRNA) was extracted from both parental and recombinant viruses using a commercial RNA extraction kit (Vazyme, China) according to the manufacturer’s instructions. A total of 12 μL of viral RNA was mixed with 4 μL of 5× loading buffer (Yeasen, China) and resolved on a 7.5% denaturing polyacrylamide gel (Epizyme, China) in 1× TBE running buffer at a constant voltage of 180 V for 4 h. The gel was stained with 0.03% nucleic acid stain (Yeasen, China) in 0.9% NaCl for 30 min post-electrophoresis. Visualization was performed using a gel imaging system.

### Growth kinetics of viruses

MA104 cells were inoculated with parental and recombinant viruses at an MOI of 0.01. Virus-containing supernatants were collected at 12-hour intervals post-infection until 60 hpi, with triplicate biological replicates at each time point. Infectivity titers, determined by Spear-Karber TCID_50_ assay, were used to generate replication curves (52).

### Plaque assay

Virus samples were serially diluted 10-fold and added to a monolayer of MA104 cells for 1 h at 37°C. After the adsorption period, 2 mL of plaque agarose mixture was added to each well. The mixture was prepared by combining equal volumes of 1.1% low-melting-point agarose (Sigma, USA), phenol red-free 2× MEM medium (Gibco, USA), 0.5 μg/mL trypsin, 1% penicillin-streptomycin (BasalMedia, China), and 0.1% DEAE-Dextran (Standard, China). The cells were incubated for 3 days. After incubation, plaques were visualized by staining with PBS containing 0.41 mg/mL Neutral Red (Sigma-Aldrich, USA). Plaque diameters were measured using ImageJ software (53).

### Active immunization in BALB/c mice

Four-week-old female BALB/c mice (Shanghai Lab Animal Research Center, China) were randomly divided into five groups (*n* = 5). The groups included three experimental groups, one negative control group, and one positive control group. Viral stocks were standardized to 10^6^ TCID_50_/mL using the Spear-Karber TCID_50_ assay for inoculation. Experimental groups received subcutaneous injections of 300 μL recombinant viruses rNJ2012-NSP1-fG5-VP7, rNJ2012-NSP3-haG4-VP7, or rNJ2012-fG5-VP7/haG4- VP7. Controls were administered either wild-type virus or PBS at equivalent volumes. Mice were immunized twice, with a 14-day interval between injections. Serum and fecal samples were collected on 0, 14, 28, and 40 dpi, with aliquots cryopreserved at −80°C for later analysis. Three weeks after the secondary immunization, all mice were euthanized by cervical dislocation under anesthesia, and then splenocytes were isolated for assessment of cellular immune responses.

### Passive immunization in BALB/c suckling mice

Four-week-old female BALB/c mice were randomly assigned into three groups (*n* = 6). Viral stocks were standardized to 10^6^ TCID_50_/mL using Spear-Karber TCID_50_ assay for inoculation. One of the groups was subcutaneously injected with 300 µL of rNJ2012-fG5-VP7/haG4-VP7, while the control groups received either wild-type virus or PBS at equivalent volumes. Mice were immunized twice, with a 14-day interval between the two doses. Following the second immunization, females were co-housed with male mice for mating, with no additional immunizations administered during the co-housing period. Five days post-birth, suckling mice, excluding those in the negative control group, were orally inoculated according to their group assignments. The inoculation was performed with 100 µL of the following viral suspensions: JSJR2023 (G4P[23], 100 μL of 5 × 10^6^ TCID_50_/mL), JSNJ2024 (G5P[7], 100 μL of 3 × 10^5^ TCID_50_/mL), or NJ2012 (G9P[7], 100 μL of 10^7^ TCID_50_/mL). All suckling mice were monitored daily for consecutive 5 dpi, with general activity recorded and fecal consistency assessed using the diarrhea scoring system. The severity of diarrhea was evaluated using the fecal scoring system as described by Boshuizen (54).

For fecal collection from 5-day-old suckling mice, the perianal area was gently stimulated with a cotton swab to induce defecation. To increase fecal yield, stimulation was repeated at 1-minute intervals as necessary. Abdominal compression was strictly avoided to prevent gastrointestinal injury or rupture. For blood collection, ether-anesthetized suckling mice were subjected to rapid incision of the cervical skin using sterile ophthalmic scissors, followed by collection of whole blood with a sterile syringe.

### Detection by indirect enzyme-linked immunosorbent assay (ELISA)

As described previously (19, 55), the ELISA plate was coated overnight at 4°C with purified P4-P[7], G4-VP7, G5-VP7, and VP7-G9 proteins (made in lab). The plates were then washed with PBST and incubated with 5% skim milk for 2 h at 37°C. Test sera (1:100 dilution) and stool suspensions (1:50 dilution) were added in triplicate to the wells (100 μL/well) and incubated for 1 h at 37°C. Levels of serum IgG or fecal IgA were detected by incubating the plates with HRP-conjugated anti-mouse IgG antibody (Solarbio, dilution 1:20,000) or HRP-conjugated anti-mouse IgA antibody (Solarbio, dilution 1:15,000) for 1 h at 37°C. The reaction was visualized using TMB substrate (Solarbio, China), and the optical density at 450 nm (OD₄₅₀) was measured.

### Neutralization assay

Mouse sera were heat-inactivated at 56°C for 30 min using a metal heating block and serially diluted twofold in DMEM. Equal volumes of diluted sera and activated viral suspension (200 TCID_50_/0.1 mL) were thoroughly mixed and incubated at 37°C for 1 h before inoculation onto MA104 monolayers. The highest serum dilution that conferred complete protection against CPE at 72 hpi was identified. Neutralizing antibody titers were computed using the Kaeber formula (52).

### Lymphocyte proliferation assay

Splenocytes were aseptically isolated from mechanically dissociated mouse spleens using a lymphocyte separation kit (TBD, China). The cells were resuspended in Roswell Park Memorial Institute 1640 medium (RPMI-1640, BasalMedia, China) supplemented with 10% FBS to a density of 10⁵ cells/well and seeded into 96-well cell culture plates. The cells were incubated for 72 h at 37°C. Lymphocyte proliferation was then assessed using a Cell Counting Kit-8 (CCK-8, Beyotime, China). All procedures were performed according to the manufacturer’s instructions.

### Cytokine detection

The concentrations of IFN-γ and IL-4 in mouse spleen lymphocyte supernatants were then quantified using commercial ELISA kits (MeiMian, China) according to the manufacturer’s instructions.

### T-cell and B-cell analysis

Isolated cells were divided into two aliquots. One aliquot was resuspended in 300 μL of PBS containing PE-conjugated anti-mouse CD4 (BioLegend, USA), APC-conjugated anti-mouse CD8a (BioLegend, USA), and FITC-conjugated anti-mouse CD3e (BD Biosciences, USA) fluorescent antibodies. The other aliquot was resuspended in 300 μL of PBS containing Percp-Cy5.5-conjugated anti-mouse B220 (BD, USA) and FITC-conjugated anti-mouse CD19 (BioLegend, USA) fluorescent antibodies. Stained cells were washed twice with PBS and analyzed using BD Accuri C6 Plus flow cytometer (BD, USA). Then, Data were subsequently analyzed using FlowJo software (BD, USA).

### Histological examination

Duodenal, jejunal, and ileal segments collected from suckling mice in each group were fixed in 10% neutral buffered formalin, processed into histopathological sections, and stained with hematoxylin and eosin. Pathological alterations were then examined under a microscope.

### Real-time quantitative PCR

Stool samples and intestinal tissues were collected from suckling mice at 24-h intervals post-challenge for RT-qPCR analysis to assess viral shedding and tissue distribution. The cDNA was extracted using a commercial RNA extraction kit and reverse transcriptase (Cowin, China) according to the manufacturer’s instructions. The resulting cDNA was then used as the template for RT-qPCR to measure the expression levels of PoRV-NSP5 using a hydrolysis probe assay. The primer sequences of PoRV-NSP5 are listed in Table S1.

### Statistical analysis

All experimental data were statistically analyzed using GraphPad Prism version 8 (GraphPad Software, USA). Student’s *t*-test and two-way ANOVA were applied as appropriate. Statistical significance was defined as *P* < 0.05 for all comparisons.

## Data Availability

All relevant data are in the article and its supplemental material.

## SUPPLEMENTAL MATERIAL

File S1 jvi.02015-25-s0001.pdf Table S1; Fig. S1 to S4.

File S2 jvi.02015-25-s0002.pdf Original WB spectra.

ASM does not own the copyrights to Supplemental Material that may be linked to, or accessed through, an article. The authors have granted ASM a non-exclusive, world-wide license to publish the Supplemental Material files. Please contact the corresponding author directly for reuse.

## References

[1] Crawford SE, Ramani S, Tate JE, Parashar UD, Svensson L, Hagbom M, Franco MA, Greenberg HB, O’Ryan M, Kang G, Desselberger U, Estes MK. 2017. Rotavirus infection. Nat Rev Dis Primers 3:17083. doi:10.1038/nrdp.2017.83 29119972 PMC5858916

[2] Vlasova AN, Amimo JO, Saif LJ. 2017. Porcine rotaviruses: epidemiology, immune responses and control strategies. Viruses 9:48. doi:10.3390/v9030048 28335454 PMC5371803

[3] Estes MK, Cohen J. 1989. Rotavirus gene structure and function. Microbiol Rev 53:410–449. doi:10.1128/mr.53.4.410-449.1989 2556635 PMC372748

[4] Matthijnssens J, Otto PH, Ciarlet M, Desselberger U, Van Ranst M, Johne R. 2012. VP6-sequence-based cutoff values as a criterion for rotavirus species demarcation. Arch Virol 157:1177–1182. doi:10.1007/s00705-012-1273-3 22430951

[5] Wenske O, Rückner A, Piehler D, Schwarz BA, Vahlenkamp TW. 2018. Epidemiological analysis of porcine rotavirus A genotypes in Germany. Vet Microbiol 214:93–98. doi:10.1016/j.vetmic.2017.12.014 29408039

[6] Lv Y, Tong Z, Liu J, Zhang Z, Wang C, Zeng Y, Liu P, Zong X, Chen G, Chen H, Tan C. 2024. Molecular characterization and pathogenicity analysis of porcine rotavirus A. Viruses 16:1842. doi:10.3390/v16121842 39772152 PMC11680200

[7] Matthijnssens J, Ciarlet M, Rahman M, Attoui H, Bányai K, Estes MK, Gentsch JR, Iturriza-Gómara M, Kirkwood CD, Martella V, Mertens PPC, Nakagomi O, Patton JT, Ruggeri FM, Saif LJ, Santos N, Steyer A, Taniguchi K, Desselberger U, Van Ranst M. 2008. Recommendations for the classification of group A rotaviruses using all 11 genomic RNA segments. Arch Virol 153:1621–1629. doi:10.1007/s00705-008-0155-1 18604469 PMC2556306

[8] Zhao B, Pan X, Teng Y, Xia W, Wang J, Wen Y, Chen Y. 2015. Rotavirus VP7 epitope chimeric proteins elicit cross-immunoreactivity in guinea pigs. Virol Sin 30:363–370. doi:10.1007/s12250-015-3620-5 26459269 PMC8200902

[9] Yin Y, Zhu L, Liu P, Zhao J, Fan Y, Sun X, Xu Z. 2019. Evaluation on the efficacy and immunogenicity of recombinant DNA plasmids expressing S gene from porcine epidemic diarrhea virus and VP7 gene from porcine rotavirus. Braz J Microbiol 50:279–286. doi:10.1007/s42770-018-0022-5 30637649 PMC6863295

[10] Liu X, Li S, Yu J, Chai P, Xie Z, Pang L, Li J, Zhu W, Ren W, Duan Z. 2024. Establishment of a reverse genetics system for rotavirus vaccine strain LLR and developing vaccine candidates carrying VP7 gene cloned from human strains circulating in China. J Med Virol 96:e70065. doi:10.1002/jmv.70065 39610277

[11] Ghonaim AH, Rouby SR, Nageeb WM, Elgendy AA, Xu R, Jiang C, Ghonaim NH, He Q, Li W. 2025. Insights into recent advancements in human and animal rotavirus vaccines: Exploring new frontiers. Virol Sin 40:1–14. doi:10.1016/j.virs.2024.12.001 39672271 PMC11962973

[12] Woode GN, Bridger J, Hall GA, Jones JM, Jackson G. 1976. The isolation of reovirus-like agents (rota-viruses) from acute gastroenteritis of piglets. J Med Microbiol 9:203–209. doi:10.1099/00222615-9-2-203 180294

[13] Tao R, Chang X, Zhou J, Zhu X, Yang S, Li K, Gu L, Zhang X, Li B. 2023. Molecular epidemiological investigation of group A porcine rotavirus in East China. Front Vet Sci 10:1138419. doi:10.3389/fvets.2023.1138419 37026094 PMC10070975

[14] Luo S, Chen X, Yan G, Chen S, Pan J, Zeng M, Han H, Guo Y, Zhang H, Li J, Mo M, Liu M, Huang L. 2022. Emergence of human-porcine reassortment G9P[19] porcine rotavirus A strain in Guangdong Province, China. Front Vet Sci 9:1111919. doi:10.3389/fvets.2022.1111919 36699335 PMC9868962

[15] Qiao M, Li M, Li Y, Wang Z, Hu Z, Qing J, Huang J, Jiang J, Jiang Y, Zhang J, Gao C, Yang C, Li X, Zhou B. 2024. Recent molecular characterization of porcine rotaviruses detected in China and their phylogenetic relationships with human rotaviruses. Viruses 16:453. doi:10.3390/v16030453 38543818 PMC10975774

[16] Zhang F, Luo Y, Lin C, Tan M, Wan P, Xie B, Xiong L, Ji H. 2024. Epidemiological monitoring and genetic variation analysis of pathogens associated with porcine viral diarrhea in southern China from 2021 to 2023. Front Microbiol 15:1303915. doi:10.3389/fmicb.2024.1303915 38572229 PMC10987963

[17] Liu H, Tian H, Hao P, Du H, Wang K, Qiu Y, Yin X, Wu N, Du Q, Tong D, Huang Y. 2024. PoRVA G9P[23] and G5P[7] infections differentially promote PEDV replication by reprogramming glutamine metabolism. PLoS Pathog 20:e1012305. doi:10.1371/journal.ppat.1012305 38905309 PMC11221755

[18] Wang J, Zhou J, Zhu X, Bian X, Han N, Fan B, Gu L, Cheng X, Li S, Tao R, Li J, Zhang X, Li B. 2024. Isolation and characterization of a G9P[23] porcine rotavirus strain AHFY2022 in China. Microb Pathog 190:106612. doi:10.1016/j.micpath.2024.106612 38467166

[19] Tang X, Li S, Zhou J, Bian X, Wang J, Han N, Zhu X, Tao R, Wang W, Sun M, Li P, Zhang X, Li B. 2024. Recombinant bivalent subunit vaccine combining truncated VP4 from P[7] and P[23] induces protective immunity against prevalent porcine rotaviruses. J Virol 98:e0021224. doi:10.1128/jvi.00212-24 38591886 PMC11092341

[20] Kanai Y, Komoto S, Kawagishi T, Nouda R, Nagasawa N, Onishi M, Matsuura Y, Taniguchi K, Kobayashi T. 2017. Entirely plasmid-based reverse genetics system for rotaviruses. Proc Natl Acad Sci USA 114:2349–2354. doi:10.1073/pnas.1618424114 28137864 PMC5338561

[21] Komoto S, Fukuda S, Kugita M, Hatazawa R, Koyama C, Katayama K, Murata T, Taniguchi K. 2019. Generation of infectious recombinant human rotaviruses from just 11 cloned cDNAs encoding the rotavirus genome. J Virol 93:e02207-18. doi:10.1128/JVI.02207-18 30728265 PMC6450123

[22] Sánchez-Tacuba L, Feng N, Meade NJ, Mellits KH, Jaïs PH, Yasukawa LL, Resch TK, Jiang B, López S, Ding S, Greenberg HB. 2020. An optimized reverse genetics system suitable for efficient recovery of Simian, human, and murine-like rotaviruses. J Virol 94:e01294-20. doi:10.1128/JVI.01294-20 32759316 PMC7459567

[23] Kanda M, Fukuda S, Hamada N, Nishiyama S, Masatani T, Fujii Y, Izumi F, Okajima M, Taniguchi K, Sugiyama M, Komoto S, Ito N. 2022. Establishment of a reverse genetics system for avian rotavirus A strain PO-13. J Gen Virol 103. doi:10.1099/jgv.0.001760 35749287

[24] Snyder AJ, Agbemabiese CA, Patton JT. 2024. Production of OSU G5P[7] porcine rotavirus expressing a fluorescent reporter via reverse genetics. Viruses 16:411. doi:10.3390/v16030411 38543776 PMC10974435

[25] Philip AA, Patton JT. 2020. Expression of separate heterologous proteins from the rotavirus NSP3 genome segment using a translational 2A stop-restart element. J Virol 94:e00959-20. doi:10.1128/JVI.00959-20 32611753 PMC7459566

[26] Zhu Y, Sánchez-Tacuba L, Hou G, Kawagishi T, Feng N, Greenberg HB, Ding S. 2022. A recombinant murine-like rotavirus with nano-luciferase expression reveals tissue tropism, replication dynamics, and virus transmission. Front Immunol 13:911024. doi:10.3389/fimmu.2022.911024 35967392 PMC9372724

[27] Kawagishi T, Sánchez-Tacuba L, Feng N, Costantini VP, Tan M, Jiang X, Green KY, Vinjé J, Ding S, Greenberg HB. 2023. Mucosal and systemic neutralizing antibodies to norovirus induced in infant mice orally inoculated with recombinant rotaviruses. Proc Natl Acad Sci USA 120:e2214421120. doi:10.1073/pnas.2214421120 36821582 PMC9992845

[28] Diebold O, Gonzalez V, Venditti L, Sharp C, Blake RA, Tan WS, Stevens J, Caddy S, Digard P, Borodavka A, Gaunt E. 2022. Using species a rotavirus reverse genetics to engineer chimeric viruses expressing SARS-CoV-2 spike epitopes. J Virol 96:e0048822. doi:10.1128/jvi.00488-22 35758692 PMC9327695

[29] Philip AA, Hu S, Dai J, Patton JT. 2023. Recombinant rotavirus expressing the glycosylated S1 protein of SARS-CoV-2. J Gen Virol 104:001899. doi:10.1099/jgv.0.001899 37830788 PMC10721933

[30] Philip AA, Patton JT. 2022. Generation of recombinant rotaviruses expressing human norovirus capsid proteins. J Virol 96:e0126222. doi:10.1128/jvi.01262-22 36314817 PMC9682992

[31] Pietrzak B, Tomela K, Olejnik-Schmidt A, Mackiewicz A, Schmidt M. 2020. Secretory IgA in intestinal mucosal secretions as an adaptive barrier against microbial cells. Int J Mol Sci 21:9254. doi:10.3390/ijms21239254 33291586 PMC7731431

[32] Ishida SI, Feng N, Gilbert JM, Tang B, Greenberg HB. 1997. Immune responses to individual rotavirus proteins following heterologous and homologous rotavirus infection in mice. J Infect Dis 175:1317–1323. doi:10.1086/516462 9180169

[33] Merchant AA, Groene WS, Cheng EH, Shaw RD. 1991. Murine intestinal antibody response to heterologous rotavirus infection. J Clin Microbiol 29:1693–1701. doi:10.1128/jcm.29.8.1693-1701.1991 1761691 PMC270186

[34] Langel SN, Paim FC, Lager KM, Vlasova AN, Saif LJ. 2016. Lactogenic immunity and vaccines for porcine epidemic diarrhea virus (PEDV): historical and current concepts. Virus Res 226:93–107. doi:10.1016/j.virusres.2016.05.016 27212686 PMC7111331

[35] Kumar D, Shepherd FK, Springer NL, Mwangi W, Marthaler DG. 2022. Rotavirus infection in swine: genotypic diversity, immune responses, and role of gut microbiome in rotavirus immunity. Pathogens 11:1078. doi:10.3390/pathogens11101078 36297136 PMC9607047

[36] Chattha KS, Roth JA, Saif LJ. 2015. Strategies for design and application of enteric viral vaccines. Annu Rev Anim Biosci 3:375–395. doi:10.1146/annurev-animal-022114-111038 25387111

[37] Hou G, Zeng Q, Matthijnssens J, Greenberg HB, Ding S. 2021. Rotavirus NSP1 contributes to intestinal viral replication, pathogenesis, and transmission. mBio 12:e0320821. doi:10.1128/mBio.03208-21 34903043 PMC8669464

[38] Valusenko-Mehrkens R, Gadicherla AK, Johne R, Falkenhagen A. 2023. Strain-specific interactions between the viral capsid proteins VP4, VP7 and VP6 influence rescue of rotavirus reassortants by reverse genetics. Int J Mol Sci 24:5670. doi:10.3390/ijms24065670 36982745 PMC10054668

[39] Blutt SE, Crawford SE, Warfield KL, Lewis DE, Estes MK, Conner ME. 2004. The VP7 outer capsid protein of rotavirus induces polyclonal B-cell activation. J Virol 78:6974–6981. doi:10.1128/JVI.78.13.6974-6981.2004 15194774 PMC421650

[40] Sharma AD, Magdaleno JSL, Singh H, Orduz AFC, Cavallo L, Chawla M. 2025. Immunoinformatics-driven design of a multi-epitope vaccine targeting neonatal rotavirus with focus on outer capsid proteins VP4 and VP7 and non structural proteins NSP2 and NSP5. Sci Rep 15:11879. doi:10.1038/s41598-025-95256-8 40195509 PMC11976959

[41] Desselberger U. 2020. What are the limits of the packaging capacity for genomic RNA in the cores of rotaviruses and of other members of the Reoviridae? Virus Res 276:197822. doi:10.1016/j.virusres.2019.197822 31783039

[42] Kotaki T, Kanai Y, Ogden KM, Onishi M, Kumthip K, Khamrin P, Boonyos P, Phoosangwalthong P, Singchai P, Luechakham T, Minami S, Chen Z, Hirai K, Tacharoenmuang R, Mizushima H, Ushijima H, Maneekarn N, Kobayashi T. 2025. A rotavirus VP4 or VP7 monoreassortant panel identifies genotypes that are less susceptible to neutralization by systemic antibodies induced by vaccination or natural infection. mBio 16:e0089725. doi:10.1128/mbio.00897-25 40444468 PMC12239578

[43] Wang Y, Yu B, Luo Y, Zheng P, Mao X, Huang Z, Yu J, Luo J, Yan H, Wu A, He J. 2023. Interferon-λ3 alleviates intestinal epithelium injury induced by porcine rotavirus in mice. Int J Biol Macromol 240:124431. doi:10.1016/j.ijbiomac.2023.124431 37060970

[44] Li Y, Wang F, Kan R, Cao H, Tang C, Yue H, Zhang B. 2022. Genetic and immunological characterization of G9 group A porcine rotaviruses in China. Zoonoses Public Health 69:694–703. doi:10.1111/zph.12958 35608375

[45] Dudek T, Knipe DM. 2006. Replication-defective viruses as vaccines and vaccine vectors. Virology (Auckl) 344:230–239. doi:10.1016/j.virol.2005.09.020 16364753

[46] Kotaki T, Kanai Y, Onishi M, Minami S, Chen Z, Nouda R, Nurdin JA, Yamasaki M, Kobayashi T. 2024. Generation of single-round infectious rotavirus with a mutation in the intermediate capsid protein VP6. J Virol 98:e0076224. doi:10.1128/jvi.00762-24 38837379 PMC11265344

[47] Kotaki T, Kanai Y, Onishi M, Sakai Y, Motooka D, Chen Z, Enoki Y, Komatsu S, Hirai K, Minami S, Kawagishi T, Ushijima H, Kobayashi T. 2025. Single-round infectious rotaviruses with deletions of VP7 or VP4 genes, based on SA11 and WC3 strain backbones, and their potential use as viral vectors. PLoS Pathog 21:e1013484. doi:10.1371/journal.ppat.1013484 40953082 PMC12435675

[48] Tao R, Cheng X, Gu L, Zhou J, Zhu X, Zhang X, Guo R, Wang W, Li B. 2024. Lipidomics reveals the significance and mechanism of the cellular ceramide metabolism for rotavirus replication. J Virol 98:e0006424. doi:10.1128/jvi.00064-24 38488360 PMC11019908

[49] Bian X, Li S, Wang J, Han N, Lu H, Cheng X, Zhou J, Tao R, Zhu X, Dong H, Zhang X, Li B. 2024. Prokaryotic expression, antibody preparation and application of major non-structural proteins of porcine rotavirus. Sci Agric Sin 57:3494–3506. doi:10.3864/j.issn.0578-1752.2024.17.014

[50] Komoto S, Fukuda S, Ide T, Ito N, Sugiyama M, Yoshikawa T, Murata T, Taniguchi K. 2018. Generation of recombinant rotaviruses expressing fluorescent proteins by using an optimized reverse genetics system. J Virol 92:e00588-18. doi:10.1128/JVI.00588-18 29669834 PMC6002737

[51] Wei J, Radcliffe S, Pirrone A, Lu M, Li Y, Cassaday J, Newhard W, Heidecker GJ, Rose Ii WA, He X, Freed D, Citron M, Espeseth A, Wang D. 2023. A novel rotavirus reverse genetics platform supports flexible insertion of exogenous genes and enables rapid development of a high-throughput neutralization assay. Viruses 15:2034. doi:10.3390/v15102034 37896813 PMC10611407

[52] Kärber G. 1931. Beitrag zur kollektiven Behandlung pharmakologischer Reihenversuche. Archiv F Experiment Pathol U Pharmakol 162:480–483. doi:10.1007/BF01863914

[53] Schneider CA, Rasband WS, Eliceiri KW. 2012. NIH Image to ImageJ: 25 years of image analysis. Nat Methods 9:671–675. doi:10.1038/nmeth.2089 22930834 PMC5554542

[54] Boshuizen JA, Reimerink JHJ, Korteland-van Male AM, van Ham VJJ, Koopmans MPG, Büller HA, Dekker J, Einerhand AWC. 2003. Changes in small intestinal homeostasis, morphology, and gene expression during rotavirus infection of infant mice. J Virol 77:13005–13016. doi:10.1128/jvi.77.24.13005-13016.2003 14645557 PMC296055

[55] Li S, Bian X, Wang J, Wang D, Zhou J, Song J, Wang W, Han N, Zhou J, Li Y, Tao R, Zhu X, Fan B, Dong H, Zhang X, Li B. 2025. VP4-specific IgA level as a correlate of neutralizing antibody and fecal shedding of porcine rotavirus infection. Vet Microbiol 304:110501. doi:10.1016/j.vetmic.2025.110501 40179488

