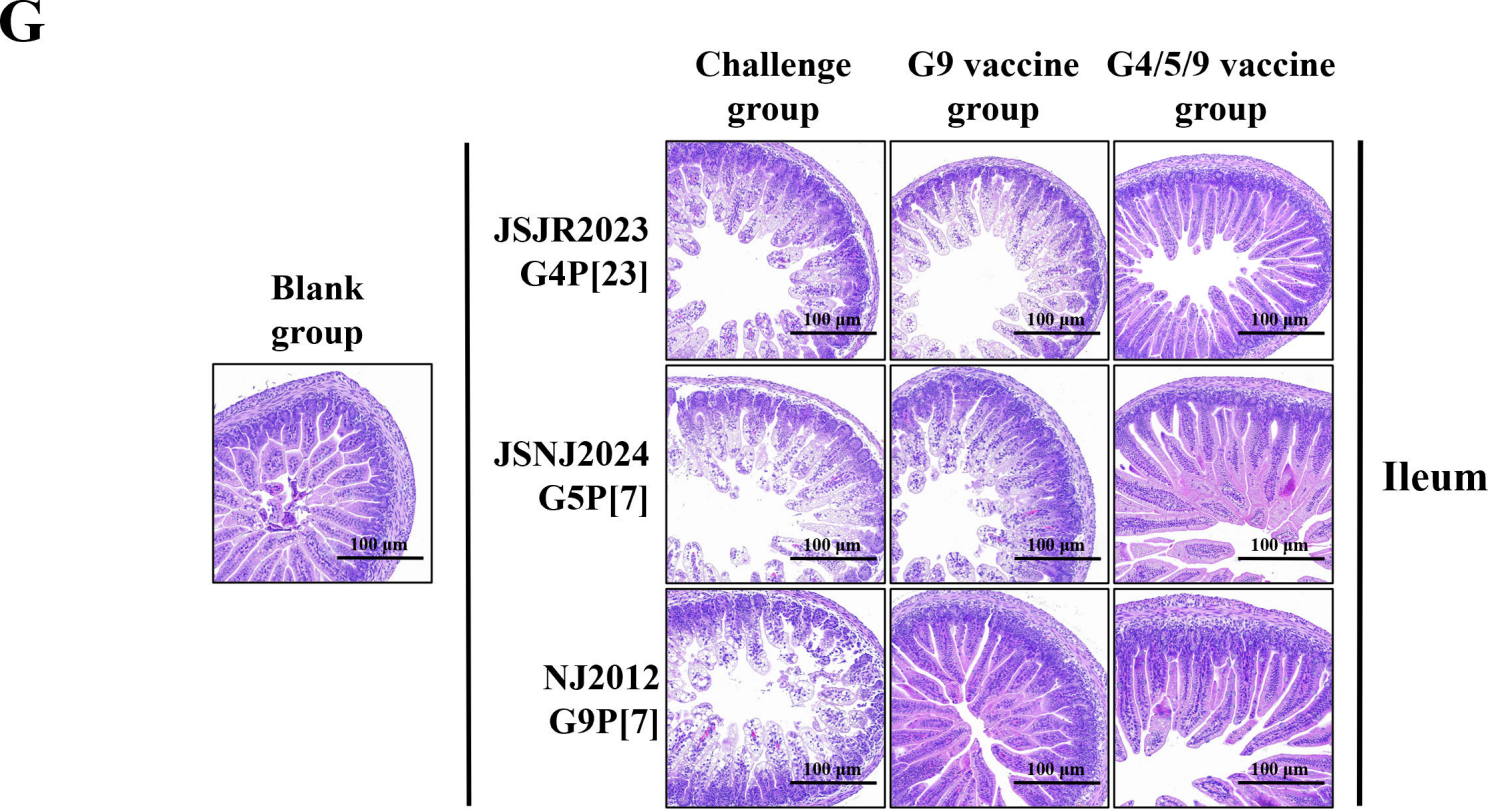# Correction for Cheng et al., “Simultaneous expression of three G genotypes of VP7 proteins in a recombinant porcine rotavirus confers protective immunity against multiple rotavirus infections”

**DOI:** 10.1128/jvi.00532-26

**Published:** 2026-06-03

**Authors:** Xi Cheng, Hui Deng, Xianyu Bian, Jianxin Wang, Chen Wang, Nan Han, Jinzhu Zhou, Xuejiao Zhu, Xuehan Zhang, Xiaojing Yang, Ran Tao, Bin Li

## AUTHOR CORRECTION

Volume 100, no. 4, e02015-25, 2026, https://doi.org/10.1128/jvi.02015-25. Figure 6D to F: the *y* axis label for all graphs should read “RNA copies/mL.”

Figure 6G should appear as shown in this correction. After publication, instances of duplication were identified in the “Blank group” column, as well as the “Challenge group” for JSNJ2024 G5P[7] and the “G9 vaccine group” for JSJR2023 G4P[23]; to resolve these concerns, a single control image is now shown, and the accidentally duplicated panel has been replaced with the correct image.

Supplemental material: In File S1, Fig. S1B should appear as shown in this correction.

**Fig 6:**