# Supplementary material for: Simultaneous expression of three G genotypes of VP7 proteins in a recombinant porcine rotavirus confers protective immunity against multiple rotavirus infections
Source: J Virol. 2026 Mar 18;100(4):e02015-25. doi: 10.1128/jvi.02015-25 (PMC13098224; doi:10.1128/jvi.02015-25)

S1 Table Primer sequences used in the experiments

Primer	Sequence (5′→3′)	Size (bp)
pT7-VP1-F	GGTTAATACGACTCACTATAGGCTATTAAAGCTGTACAATGG	3,302
pT7-VP1-R	TGGAGATGCCATGCCGACCCGGTCACATCTAAGCACTCTA	
pT7-VP2-F	GGTTAATACGACTCACTATAGGCTATTAAAGGCTCAATGG	2,717
pT7-VP2-R	TGGAGATGCCATGCCGACCCGGTCATATCTCCACAGTGG	
pT7-VP3-F	GGTTAATACGACTCACTATAGGCTATTAAAGCAGTACTAG	2,591
pT7-VP3-R	TGGAGATGCCATGCCGACCCGGTCACATCATGACTAGTGTG	
pT7-VP4-F	GGTTAATACGACTCACTATAGGCTATAAAATGGCTTCG	2,360
pT7-VP4-R	TGGAGATGCCATGCCGACCCGGTCACATCCTTTAGAA	
pT7-VP6-F	GGTTAATACGACTCACTATAGGCTTTTAAACGAAGTCTTCG	1,356
pT7-VP6-R	TGGAGATGCCATGCCGACCCGGTCACATCCTCTCACTATA	
pT7-VP7-F	GGTTAATACGACTCACTATAGGCTTTAAAAGAAGGAATTTCCG	1,062
pT7-VP7-R	TGGAGATGCCATGCCGACCCGGTCACATCATACAATTCTAACC	
pT7-NSP1-F	GGTTAATACGACTCACTATAGGCTTTTTTATGAAAAGTCTTGTGG	1,567
pT7-NSP1-R	TGGAGATGCCATGCCGACCCGGTTCACATTTTATGCTGCC	
pT7-NSP2-F	GGTTAATACGACTCACTATAGGCTTTTAAAGCGTCTCAGT	1,059
pT7-NSP2-R	TGGAGATGCCATGCCGACCCGGTCACATAAGCGCTTTCTATTC	
pT7-NSP3-F	GGTTAATACGACTCACTATAGGCTTTTAATGCTTTTCAGTGG	1,076
pT7-NSP3-R	TGGAGATGCCATGCCGACCCGGTCACATAACGCCCCTA	
pT7-NSP4-F	GGTTAATACGACTCACTATAGGCTTTTAAAAGTTCTGTTCCG	750
pT7-NSP4-R	TGGAGATGCCATGCCGACCCGGTCACATTAAGACCATTC	
pT7-NSP5-F	GGTTAATACGACTCACTATAGGCTTTTAAAGCGCTACAG	667
pT7-NSP5-R	TGGAGATGCCATGCCGACCCGGTCACAAAACGGGAGTG	
p3E5-F	GGGTCGGCATGGCATCTCCA	3,300
p3E5-R	TATAGTGAGTCGTATTAACC	
RV-VP4-F	ATGGCTTCDCTCATTTAYAGACA	1,080
RV-VP4-R	GCTTGAGAATCRTCCCARTA	
RV-VP7-F	GGCTTTAAAAGAGAGAATTTCCGTCTGG	395
RV-VP7-R	ACTGATCCTGTTGGCCATCCTTT	
qNSP5-F	CTGCTTCAAACGATCCACTCAC	
qNSP5-R	TGAATCCATAGACACGCC	
qNSP5-probe	FAM-TCGAATGCAGTTAAGACAAATGCAGACGCT-BHQ1	

FIG S1 Validation of the protein expressions in rNJ2012-NSP3-UnaG or rNJ2012-NSP3-NLuc infected MA104 cells. (**A**) Western blotting Analysis. Protein expression of RV-VP6, Flag, NLuc, and GAPDH was detected by the specific antibodies. (**B**) IFA. The cells were stained with antibodies specific to RV-VP6 (red), Flag (green), NLuc (green), and DAPI (blue). Scale bar: 50 μm.

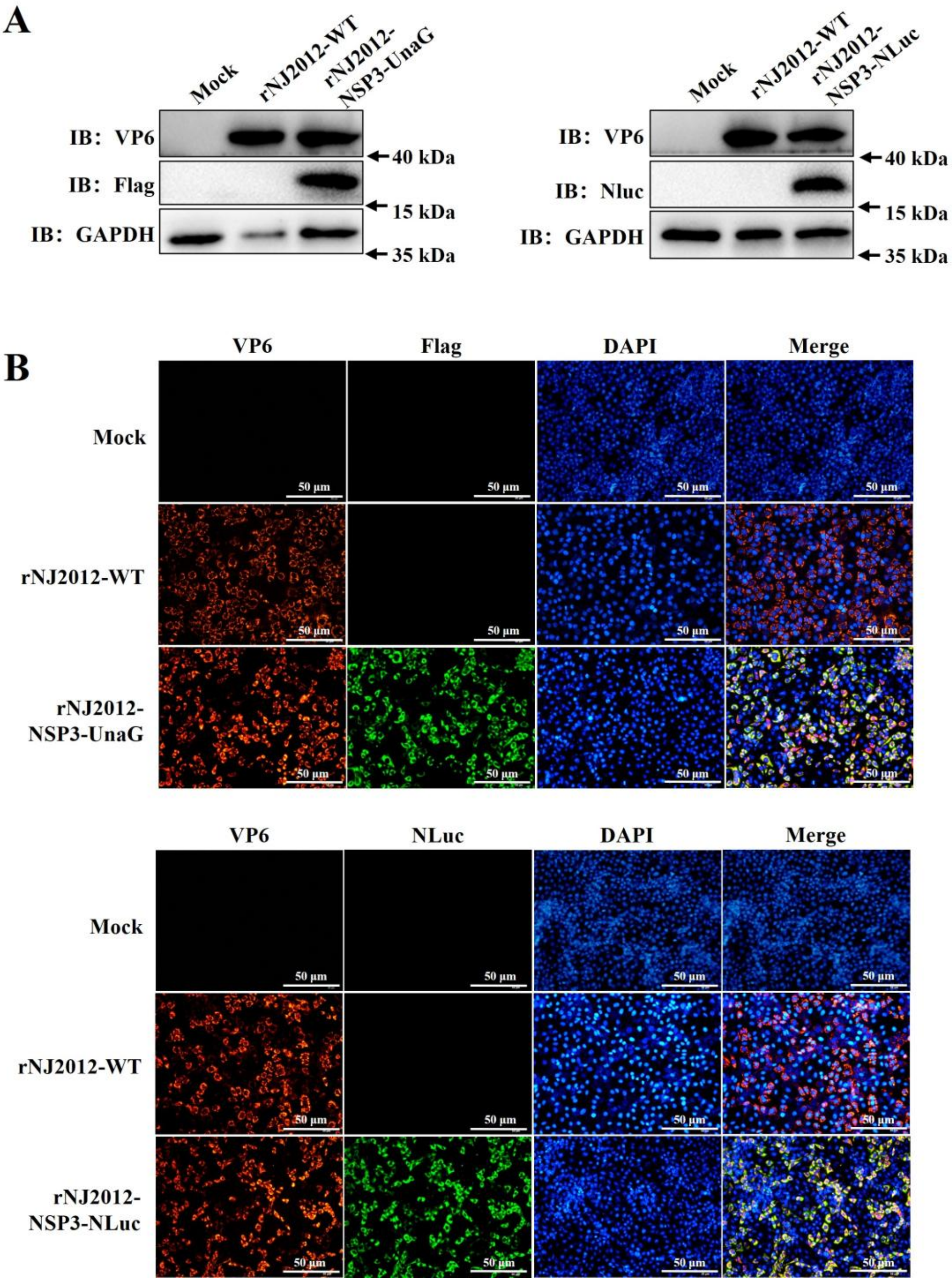

FIG S2 IFA of the protein expressions in rNJ2012-NSP1-fG5-VP7, rNJ2012-NSP3-haG4-VP7, and rNJ2012-fG5-VP7/ haG4-VP7 infected MA104 cells. (**A**) The cells were stained with antibodies specific to Flag (red), HA (green), and DAPI (blue). (**B**) The cells were stained with antibodies specific to RV-VP6 (red) and DAPI (blue). Scale bar: 50 μm.

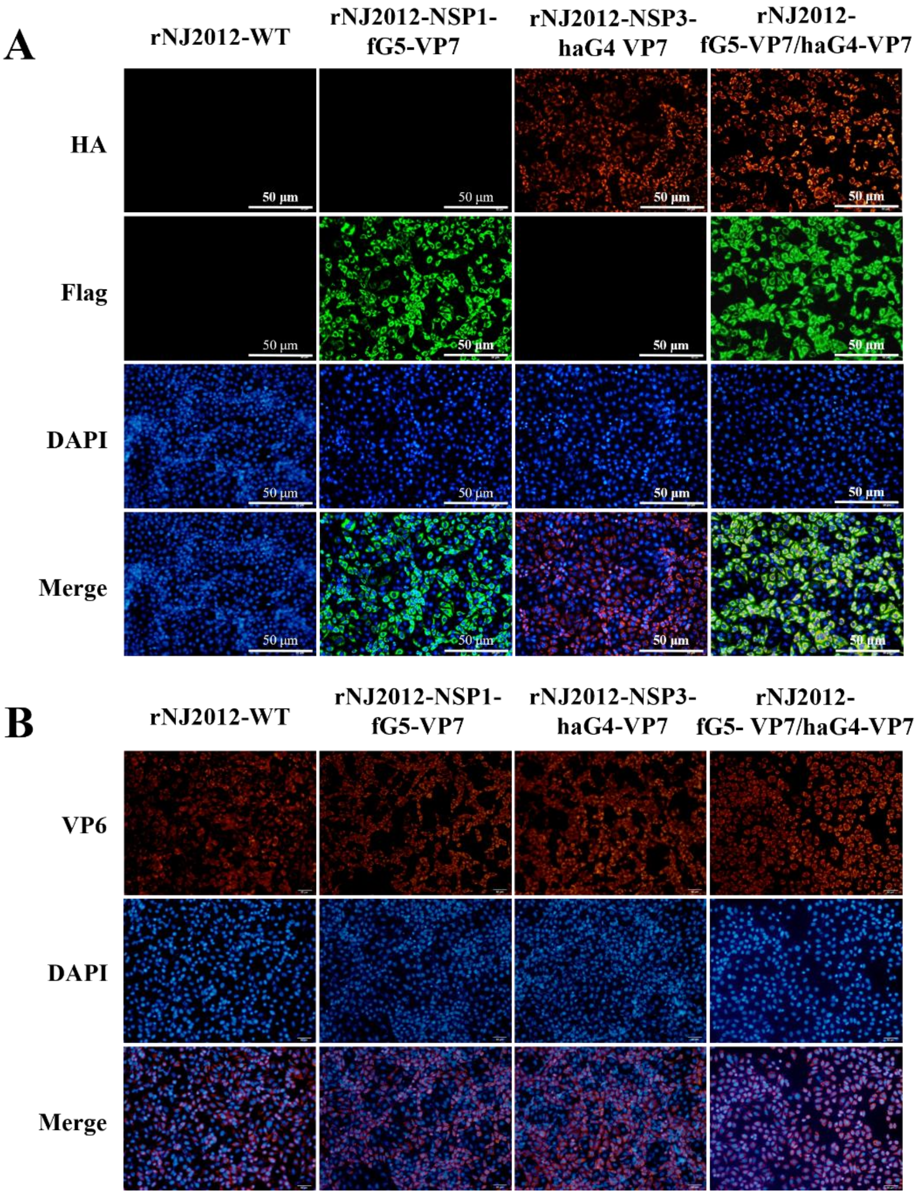

FIG S3 Genetic stability of rNJ2012-NSP1-fG5-VP7，rNJ2012-NSP3-haG4-VP7, and rNJ2012-fG5-VP7/haG4-VP7. The migrations of modified genome segment 5 and genome segment 7 are indicated by a red asterisk. Genome segments 1–11of recombinant viruses are indicated on the left side of the panels.

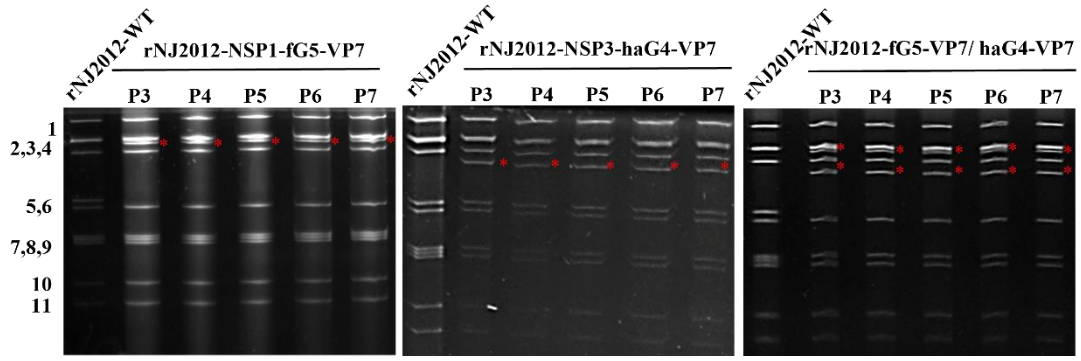

Fig S4 Flow cytometric analysis of the proportions of $CD3^{+}CD4^{+}$ T cells (**A**), $CD3^{+}CD8^{+}$ T cells (**B**), and B cells (**C**) within murine splenocytes.

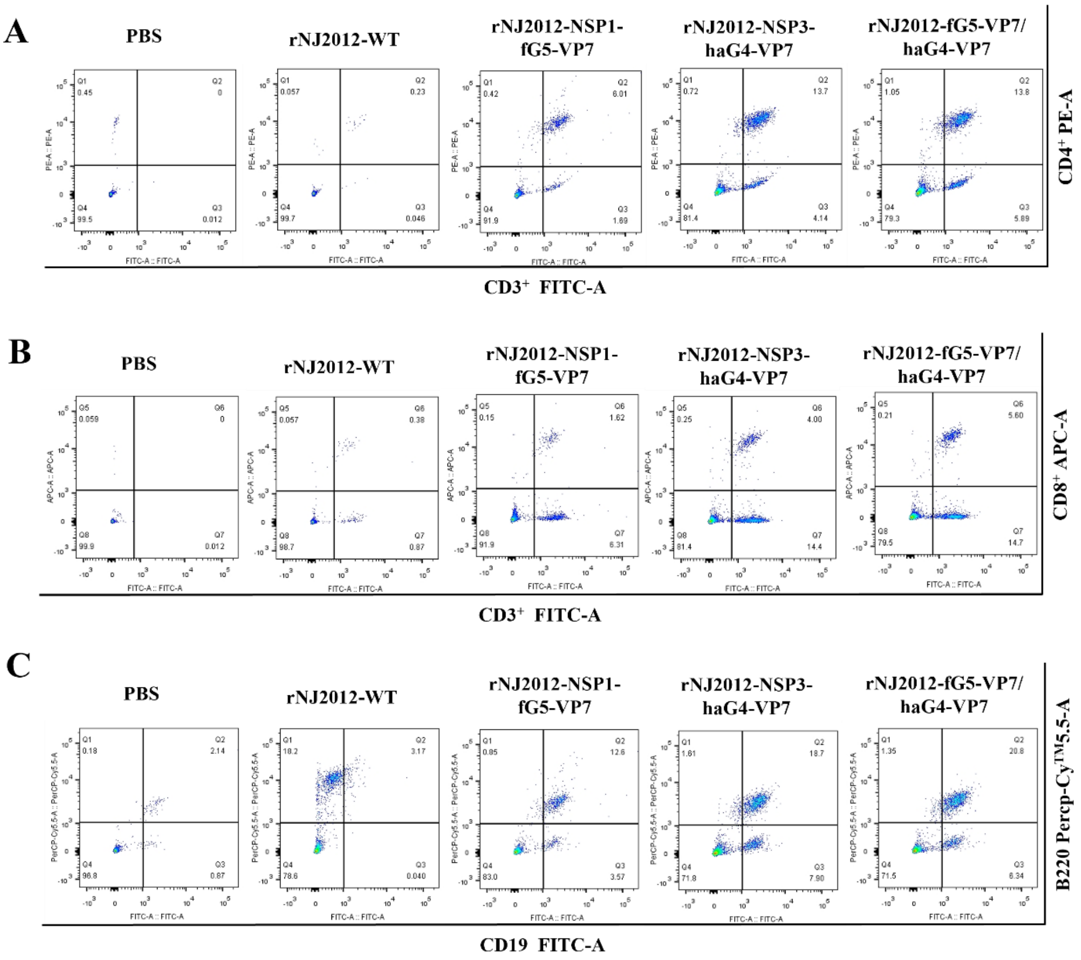

Supplement: File S1 — Table S1; Fig. S1 to S4. [file jvi.02015-25-s0001.pdf]
