# Supplementary figures and images for: Simultaneous expression of three G genotypes of VP7 proteins in a recombinant porcine rotavirus confers protective immunity against multiple rotavirus infections
Source: J Virol. 2026 Mar 18;100(4):e02015-25. doi: 10.1128/jvi.02015-25 (PMC13098224; doi:10.1128/jvi.02015-25)

**Fig 1D**

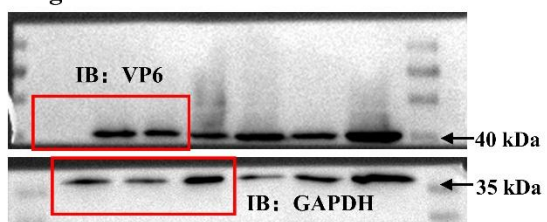

**Fig 2H**

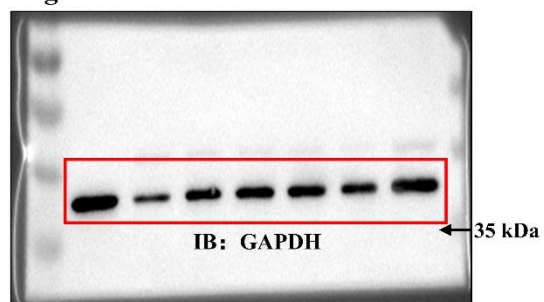

**Fig 2G**

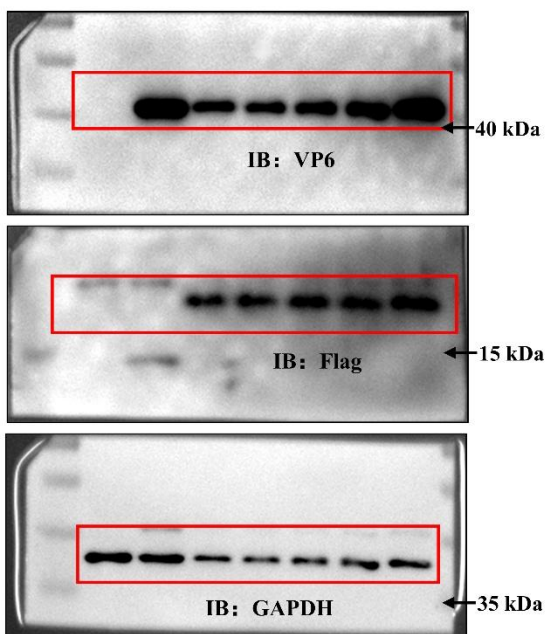

**Fig 3B**

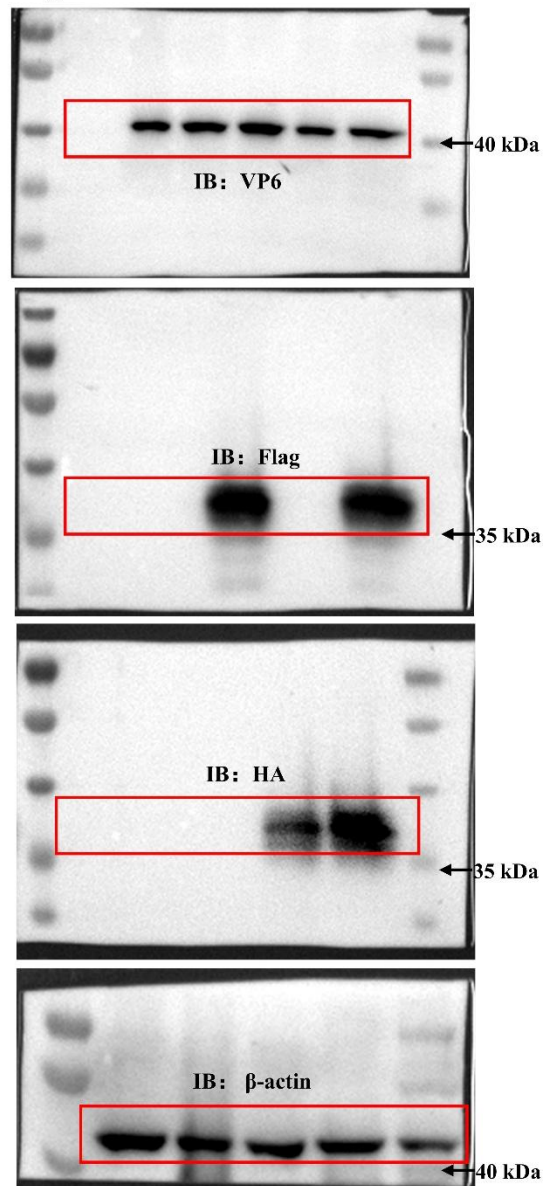

**Fig 2H**

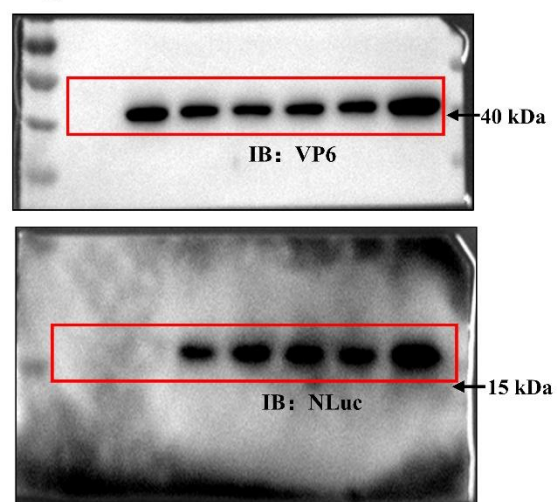

**Fig 3F**

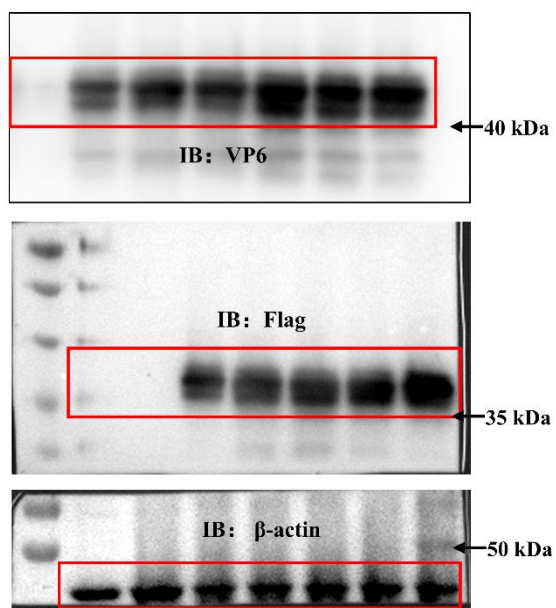

**Fig 3H**

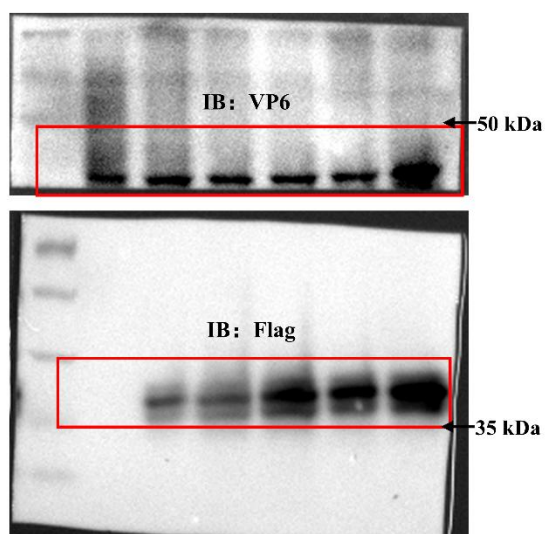

**Fig 3G**

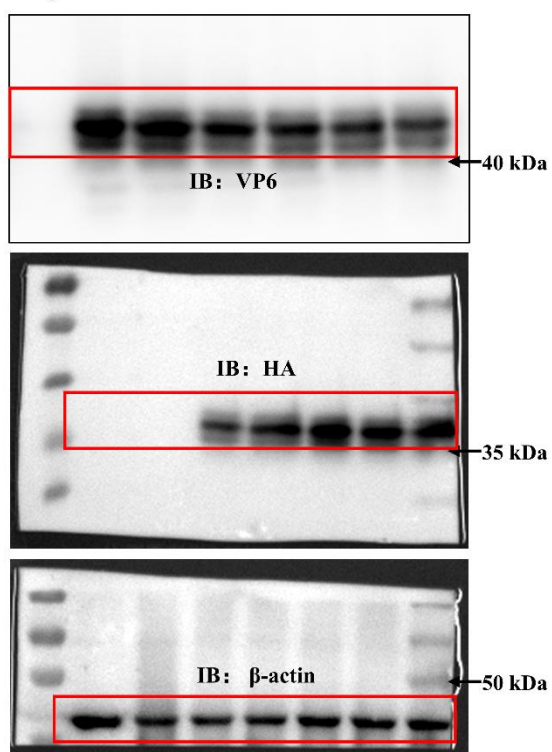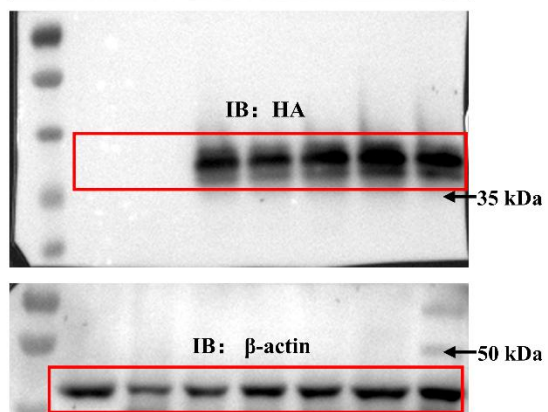

S1 Fig

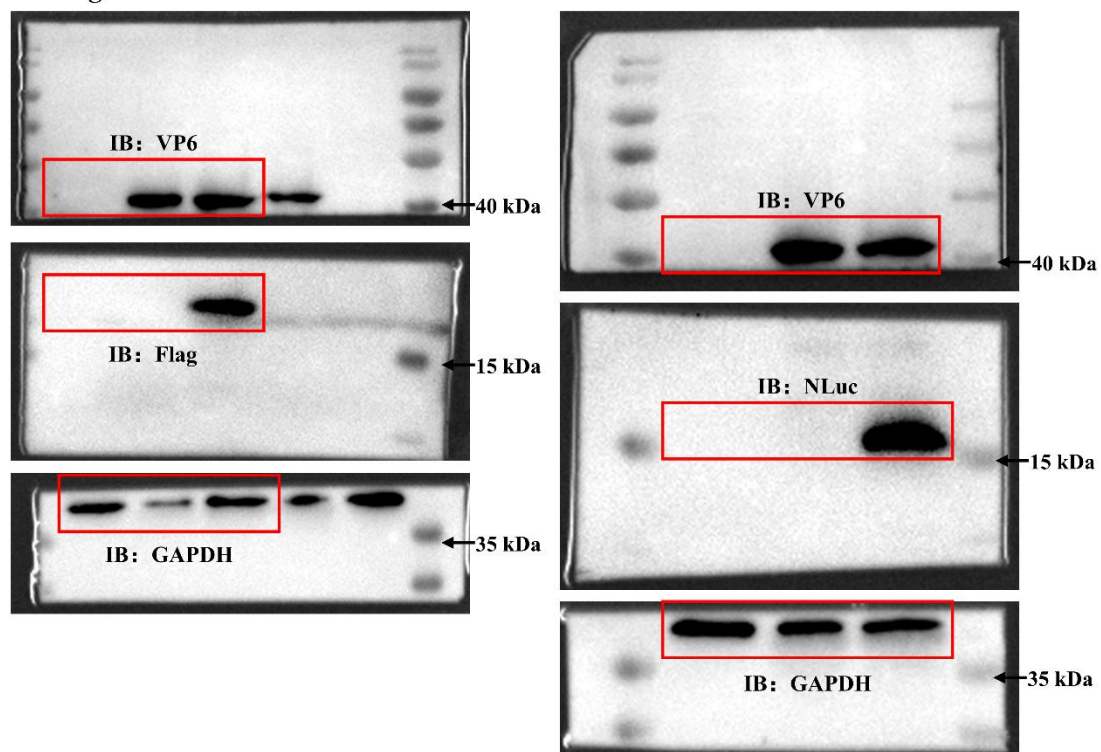

Supplement: File S2 — Original WB spectra. [file jvi.02015-25-s0002.pdf]
